# Mutations in two-component signaling systems drive experimental evolution of tigecycline and colistin resistance in *Acinetobacter baumannii*

**DOI:** 10.1128/aac.00809-25

**Published:** 2025-10-29

**Authors:** J. E. Kent, M. Elane, S. Leyn, J. Zlamal, N. Wong, M. Aizin, C. Zampaloni, S. Louvel, A. Haldimann, M. Vercruysse, A. Osterman

**Affiliations:** 1 Sanford Burnham Prebys Medical Discovery Institute 4355, La Jolla, California, USA; 2 Roche Pharma Research and Early Development, Infectious Diseases Therapeutic Area, Roche Innovation Center Basel, F. Hoffmann-La Roche 1529 , Basel, Switzerland; Universita degli Studi di Roma "La Sapienza", Rome, Italy

**Keywords:** ESKAPE pathogens, antibiotics, experimental evolution, resistance

## Abstract

The treatment of infections by *Acinetobacter baumannii*, a clinically significant nosocomial gram-negative bacterial pathogen, is hampered by antibiotic resistance, which is exacerbated by its exceptional genetic plasticity. Knowledge of the dynamics and mechanisms underlying the acquisition of antibiotic resistance is essential for the proper stewardship of their utilization. Here, we used a continuous culture device (morbidostat) to characterize the evolutionary trajectories of two *A. baumannii* strains in response to the increasing pressure of last-resort drugs, tigecycline and colistin. This approach allows us to confidently and comprehensively map resistance-driving mutations while circumventing both the “driver vs passenger” uncertainty and “selection bottleneck” limitations characteristic of clinical isolate analysis and conventional laboratory evolution, respectively. Tigecycline resistance predominantly occurred through the combination of missense mutations in the *adeSR* two-component system and disruptive events in the S-adenosyl methionine (SAM)-dependent methyltransferase, *trm*, while colistin resistance predominantly occurred through missense mutations in the gene cluster responsible for lipid A phosphoethanolamine modification, *pmrCAB*. Mapping of these mutational events over numerous publicly available *A. baumannii* genomes identified a relatively low prevalence of resistance to these two drugs. This work represents an initial step toward predictive resistomics of *A. baumannii*, leveraging gene-level genomic variations in addition to the conventional approaches based on the presence or absence of antibiotic resistance genes.

## INTRODUCTION

*Acinetobacter baumannii* is a clinically significant nosocomial gram-negative bacterial pathogen causing devastating and poorly curable infectious disease (1). The treatment of *A. baumannii* infections is severely hampered by the acquisition of antibiotic resistance (2), which is exacerbated by the exceptional genetic diversity and plasticity characteristic of this species (3, 4). Widespread carbapenem-resistant *A. baumannii* (CRAB) represents a well-known challenge (5, 6), reflecting a long and convoluted clinical history of beta-lactam antibiotics. However, recently, a growing number of *A. baumannii* clinical isolates displayed resistance to last-resort drugs, colistin (COL) and tigecycline (TGC) (7–11), prompting the development of new classes of antibiotics against this deadly pathogen (12). A comprehensive mapping of genetic features underlying susceptibility and resistance of *A. baumannii* to a broad range of established and novel antibiotics (combined with the accessibility of whole-genome sequencing) opens the opportunity to overcome the problem of preexisting or rapidly acquired antimicrobial resistance (AMR) via a rational selection of drugs and combinations thereof.

The resistome of *A. baumannii* comprises both the antibiotic resistance genes (ARGs), which commonly represent an accessory (open) part of the pan-genome (13) (i.e., outside of the core genome), and resistance-driving mutational events that alter the functions of core genes, effectively converting them into ARGs. The dissemination of ARGs, the majority of which are associated with efflux pump and β-lactamase coding genes (3), is driven by horizontal gene transfer (HGT), notably from *A. baumannii* to other bacterial pathogens (14). Major resistance mechanisms driven by mutations and IS-element insertions in *A. baumannii* were the subject of numerous mechanistic and genomic studies for all clinically relevant antibiotics, including TGC and COL (15). Genomics-driven mutational studies are typically based on sequencing of (i) drug-resistant clinical isolates or (ii) mutants obtained by conventional laboratory selection techniques. While a powerful aspect of the first strategy is its clinical relevance, its most significant limitation lies in the difficulty of distinguishing acquired mutations from neutral variations. This is particularly challenging for *A. baumannii* due to its exceptional genetic plasticity. A confident identification of an acquired (and thus, potentially resistance-driving) mutation in any gene would require the knowledge of the parental sequence, which is rarely available for clinical isolates. Laboratory approaches based on selecting resistant clones are free from this limitation but are commonly limited by selection bottlenecks and tend to yield narrow sets of mutational variants, often those of relatively low fitness and questionable clinical relevance. Moreover, neither approach provides any dynamic metrics or additional criteria for the prioritization of potential driver over passenger gene variants.

A methodology combining a continuous culturing device (morbidostat [16]) with high-coverage whole-genome sequencing (WGS) of evolving bacterial populations in paralleled time series allows us to overcome most of these limitations. Previously, we have demonstrated the utility of a morbidostat-based workflow for the experimental evolution and comparative resistomics of several established and experimental antibiotics in *A. baumannii*, *Pseudomonas aeruginosa*, and *Escherichia coli* (12, 17–19). In the current study, we have applied this established workflow to map the mutational landscapes and evolutionary trajectories of two American Type Culture Collection (ATCC) *A. baumannii* strains (ATCC 17978 and ATCC BAA-747) toward resistance to each of the two last-resort drugs, TGC and COL. The utility of deduced mutational signatures for genome-based predictive resistomics is illustrated by the analysis of ~10,000 *A*. *baumannii* genomes, which yielded initial mapping of the respective resistant variants.

Overall, this study confirmed the utility of morbidostat-based comparative resistomics for elucidating the dynamics and mechanisms of antibiotic resistance.

## MATERIALS AND METHODS

### Bacterial strains and media

Bacterial strains used in this study, *A. baumannii* ATCC 17978 and *A. baumannii* ATCC BAA-747 (herein *A. baumannii* BAA-747), were obtained from ATCC. An *A. baumannii* ATCC 17978 clone with the gene encoding the putative S-adenosyl methionine (SAM)-dependent methyltransferase, *trm*, deleted was engineered as described in Supplementary Methods. A cation-adjusted Mueller-Hinton broth (CA-MHB) with 2% dimethyl sulfoxide (DMSO) was used as a base media for the experimental evolution in the morbidostat and for MIC measurements. For morbidostat runs, the autoclaved antifoam SE-15 (Sigma) was added to a final dilution of 1/2,500.

### Evolution of antibiotic resistance

The experimental evolution of resistance towards either TGC or COL for *A. baumannii* strains ATCC 17978 and BAA-747 was performed using the custom-engineered morbidostat device as described previously (17, 19) (see Supplementary Methods). Briefly, five evolutionary runs were performed by inoculating six reactors with independently prepared log-phase starter cultures (OD_600_ = 0.02) and increasing the concentrations of the target drug (either TGC or COL) within the reactors depending on the performance of the cultures through dilutions with computer-controlled mixtures of drug-free and drug-containing media. Samples (10 mL) were collected at approximately 24-hour intervals from which cell pellets and glycerol stocks were prepared for subsequent use in DNA isolation and clonal isolation, respectively.

### Whole-genome sequencing and analysis

Genomic DNA extraction, WGS library preparation, sequencing, and sequence analysis methodology for variant calling in clonal and population samples were performed as previously described (17).

### MIC measurements

MIC values were measured for the unevolved parental clones (*A. baumannii* BAA-747 and *A. baumannii* ATCC 17978), evolved clones, and CLSI-recommended QC strains (*E. coli* ATCC 25922 and *P. aeruginosa* ATCC 27853) using the broth dilution method as described previously (17), with the same growth media base as in the morbidostat-based experiments. The fresh colonies were resuspended in freshly prepared CA-MHB medium and inoculated into 96-well plates containing twofold increasing concentrations of each compound. Measurements were performed by constant growth or end-point monitoring of OD_600_ in a BioTek ELx808 plate reader at 37°C. MICs were measured for colistin (COL), meropenem, sulbactam, tigecycline, ciprofloxacin, gentamicin, ceftazidime, cotrimoxazole (trimethoprim/ sulfamethoxazole), minocycline, levofloxacin, and tobramycin following CLSI guidelines (20).

### RT-qPCR for gene expression analysis

For RT-qPCR experiments, selected TGC^R^ or COL^R^ clones of *A. baumannii* ATCC 17978, as well as the respective unevolved parental strains, were grown overnight in CA-MHB to a density of ~2  ×  10^8^ CFU/mL in triplicate (no breakpoint for TGC^R^ is defined; therefore, a fold change of 2× relative to the parental strain was included in this classification) (COL^R^ is defined as an MIC ≥2 µg/mL). RNA isolation from flash-frozen pellets followed by RT-qPCR using *adeA*, *pmrC*, and *gyrB*-specific primers was performed as described in (17). Detailed methodology can be found in the Supplementary Methods.

### 3D modeling and genomic distribution of variations in TCS amino acid sequences

Structural models for all individual proteins and protein complexes were created using ColabFold v1.5.5 under default settings (21). To assess positional variations, relevant amino acid sequences of TCS proteins (AdeA, AdeB, PmrA, and PmrB) represented by respective global protein families from ~10,000 high-quality *A. baumannii* genomes were downloaded from the BV-BRC database along with metadata of interest (geography and multilocus sequence typing [MLST]) (22). MAFFT was used to construct multiple alignments for all collected sequences in a family. The alignment was filtered to include only sequences with ≥80% identity to the respective protein from *A. baumannii* ATCC 17978 and to retain only one best hit per genome. For each original position of the amino acid sequence of the protein from ATCC 17978 strain, Shannon entropy (23) was calculated using the formula: ∑(p*log2(p)), where *p* represents the proportion of counts for each observed amino acid in the alignment position divided by the number of aligned sequences excluding gaps and “X” symbols.

The distribution of variants of interest across both geography and MLST was assessed by comparing the overall distribution of each of these metadata categories (from BV-BRC database) for all downloaded sequences to the distributions of these categories for sequences containing only the variants of interest.

## RESULTS

### Evolution of tigecycline resistance

Two morbidostat runs of the two *A. baumannii* strains, ATCC 17978 and BAA-747, with tigecycline (TGC) were performed, and the results were analyzed as previously described (17, 19). The workflow outlined in Fig. S1A and B include four major steps: First, the cultures of each strain were the subject of continuous growth in six parallel bioreactors of a custom-engineered morbidostat instrument over 99 and 152 h, respectively. During the run, the cultures were regularly diluted with drug-free or drug-containing media depending on the observed growth rate/adaptation to gradually increasing TGC concentrations—up to 20–32 mg/L (in the first 2 to 3 days), and then to 100–320 mg/L. Of the periodically collected samples of evolving bacterial populations, 24 samples from the ATCC 17978 run (four time points: 24, 48, 80, and 99 h from six reactors) and 25 samples from the BAA-747 run (five time points: 23, 44, 68, 94, 128, and 152 h from five reactors) were analyzed by Illumina WGS. In the second step, genomic libraries representing all these samples were sequenced at 500–1,000× genomic coverage. The sequencing data were mapped to reference genomes to identify single-nucleotide variants (SNVs), short indels, and copy number variants (CNVs), allowing tentative mapping of large deletions and amplifications. IS element insertion events were mapped using the specialized software iJUMP. Mapping and assessment of the relative abundance of all mutational events were performed using the established computational pipeline as previously described and briefly outlined in Supplementary Methods (17, 19). Upon the identification and ranking of mutational events in potential driver genes (Table 1; Table S1A and B) by a combination of criteria (described in Supplementary Methods), we isolated and analyzed a panel of representative clones from retained samples at each time point using a selection optimization strategy also described in Supplementary Methods. Mutations (mapped by WGS at the third step of the workflow) and MIC^TGC^ values (determined by broth microdilution method at the fourth step) for non-redundant sets of 12 and 15 clones in ATCC 17978 and BAA-747 strain background, respectively, are provided in Table S1C and D. Overall, we observed a moderate increase in MIC^TGC^ values from 0.3125 µg/mL for both unevolved strains to a maximum of 10 µg/mL in selected mutant clones.

**TABLE 1:** Major mutational variants detected during experimental evolution of TGC resistance in *A. baumannii* strains ATCC 17978 and BAA-747^*c*^^,^^*d*^

Gene	ATCC 17978			BAA-747			Short functional description
	Variants	A_MAX_^*a*^	N_ALL_^*b*^	Variants	A_MAX_^*a*^	N_ALL_^*b*^	
AdeSR two-component signaling (TCS) system: transcriptional regulation of AdeAB efflux pump							
*adeS*	K2I; S8R; A37T; A37Glu; D60Y; **D60G**; **D60V**; A94V; **S101G**; N125S; A148V; T153M; G160S; D167N; F170I; G318S; **G318D**	**95%**	**20**	**Y31H**; (T54_W61)dupl; L133I; **A148V**; R152K; T153M; G160S; **D167A**; R313K; **G318S; G318D**	**99%**	**15**	TCS sensor histidine kinase AdeS
*adeR*	**Y31F**; D23Y	**69%**	**2**	A91V	**63%**	**2**	TCS transcriptional activator AdeR
*adeA*	*us_IS*	**73%**	**2**				Membrane protein AdeA of AdeABC efflux pump
Uncharacterized methyltransferase Trm							
*trm*	Q55fs; **N104fs**; G194R; H262Y; *IS*	**87%**	**26**	I52fs; K63fs; **Q64fs**; N113fs; L195^*d*^; Q325fs; **K392fs^*e*^**; *IS^f^*	**98%**	**21**	SAM-dependent methyltransferase
Transcriptional regulation of AdeIJK efflux pump							
*adeN*	*IS*	**45%**	**10**	*IS*	**91%**	**4**	Transcriptional regulator AdeN
Other potentially significant variants							
*abeM*	*us_IS*	**94%**	**3**				Na+-driven multidrug efflux pump of MATE family
*rpoB*				**T572I**; **G466V**; D862G	**82%**	**3**	DNA-directed RNA polymerase beta subunit
*rpoC*				∆(G1041-D1049)	34%	**1**	DNA-directed RNA polymerase beta′ subunit
*nusA*				R109C; F365S; ∆(L387-Glu388); **K480fs**	**92%**	**4**	Transcription termination protein NusA
*wbpO*				*IS; us_IS*	**71%**	**5**	UDP-N-acetyl-D-glucosamine 6-dehydrogenase
*rpsJ*				**V57L**; L102F	68%	**2**	SSU ribosomal protein S10p
*lpsC*				*IS*	**54%**	**4**	LPS core biosynthesis glycosyl transferase

^*a*^ A_MAX_: maximal relative abundance (%) reached by a variant in at least one sample.

^*b*^ N_ALL_: number of independent occurrences of a variant across all reactors.

^*c*^ Boldface type indicates variants reaching A_MAX_ ≥ 50%.

^*d*^ stop.

^*e*^ fs, frameshift.

^*f*^ IS, insertion of a mobile element (*us_IS*, insertion in the upstream of a respective gene).

**Fig 1:**
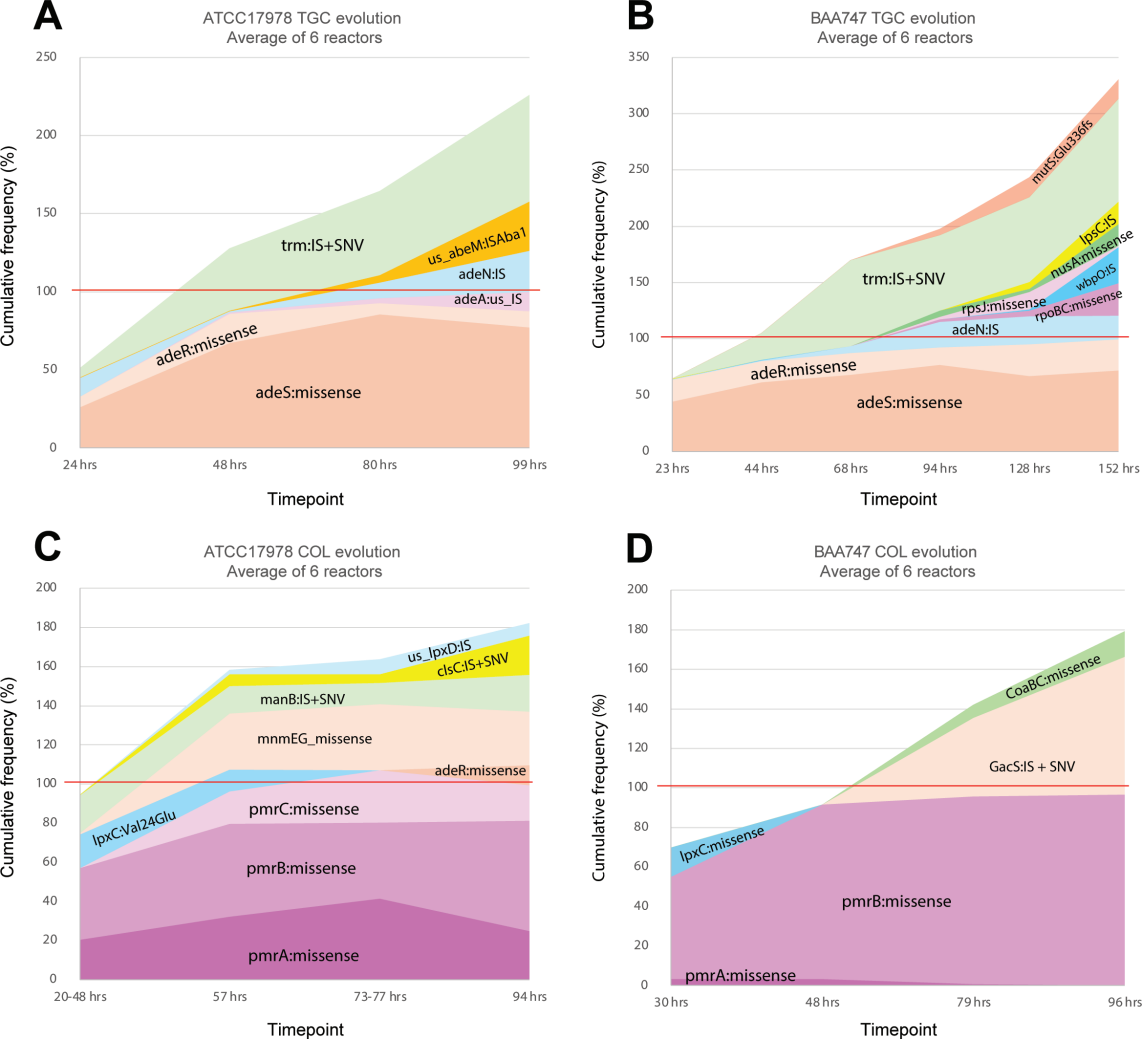
Dynamics of major mutational variants in morbidostat-based evolution of resistance against TGC (**A, B**) and COL (**C, D**) in *A. baumannii* strains ATCC 17978 (**A, C**) and BAA-747 (**B, D**). Cumulative area plots are shown for potentially significant mutational variants (SNVs and IS insertions) observed in the evolving bacterial populations. Relative abundances of selected variants (%)—averaged over all reactors and summed up for the respective genes—are plotted vs evolution (sampling) time (hours). Gene names and corresponding functions are outlined in Tables 1 and 2. The red line (at 100% corresponding to an average of one mutation per genome) emphasizes the emergence of double and triple mutants.

Both morbidostat runs resulted in complex evolutionary trajectories, characterized by the accumulation of multiple genomic variants, including point mutations, short indels, insertion sequence (IS) insertions, and deletions or amplifications of genomic loci (Table S1A and C). Major potential resistance drivers are listed in Table 1, and the dynamics of their occurrence are graphically depicted in Fig. S2A and B, showing individual reactors, and in averaged form in Fig. 1A and B. Across the two runs, a total of 28 non-redundant evolved clones (12 from ATCC 17978 and 16 from BAA-747) were isolated from selected population samples and characterized (Table S1B and D).

The evolution of TGC resistance in the two *A. baumannii* strains was marked by highly consistent mutational profiles across the reactors. A clear mechanism involving two genetic drivers for the evolution of moderate TGC resistance was captured, involving the overexpression of *adeAB* (efflux pump subunits) through missense mutations in *adeSR* (two-component transcriptional regulator), followed or preceded by disruptive mutations/IS element insertions in *trm* (SAM-dependent methyltransferase; putatively involved in ribosomal RNA methylation). In the early stage of evolution (during the first 24 h, while the drug concentration in the reactors did not exceed 2× MIC of the unevolved strains), bacterial populations were dominated by a variety of missense mutations in *adeSR* genes (Fig. 1A and B). In the middle of the run (40–80 h), a relative abundance of disruptive SNVs and IS element insertions in *trm* became comparable with the abundance of *adeSR* missense variants. The total abundance of these variants suggested a dominance in the population of dual mutational variants (AdeSR:missense+Trm:[SNV or IS]). By the end of evolutionary runs (>90 h), mutational variants in additional genes (listed in Table 1) emerged and accumulated at an average level of 10–30%. Although the functional significance of most of them remains uncertain, their accumulation (in addition to primary driver variants) suggests that they may increase the fitness under substantial drug pressure.

**Fig 2:**
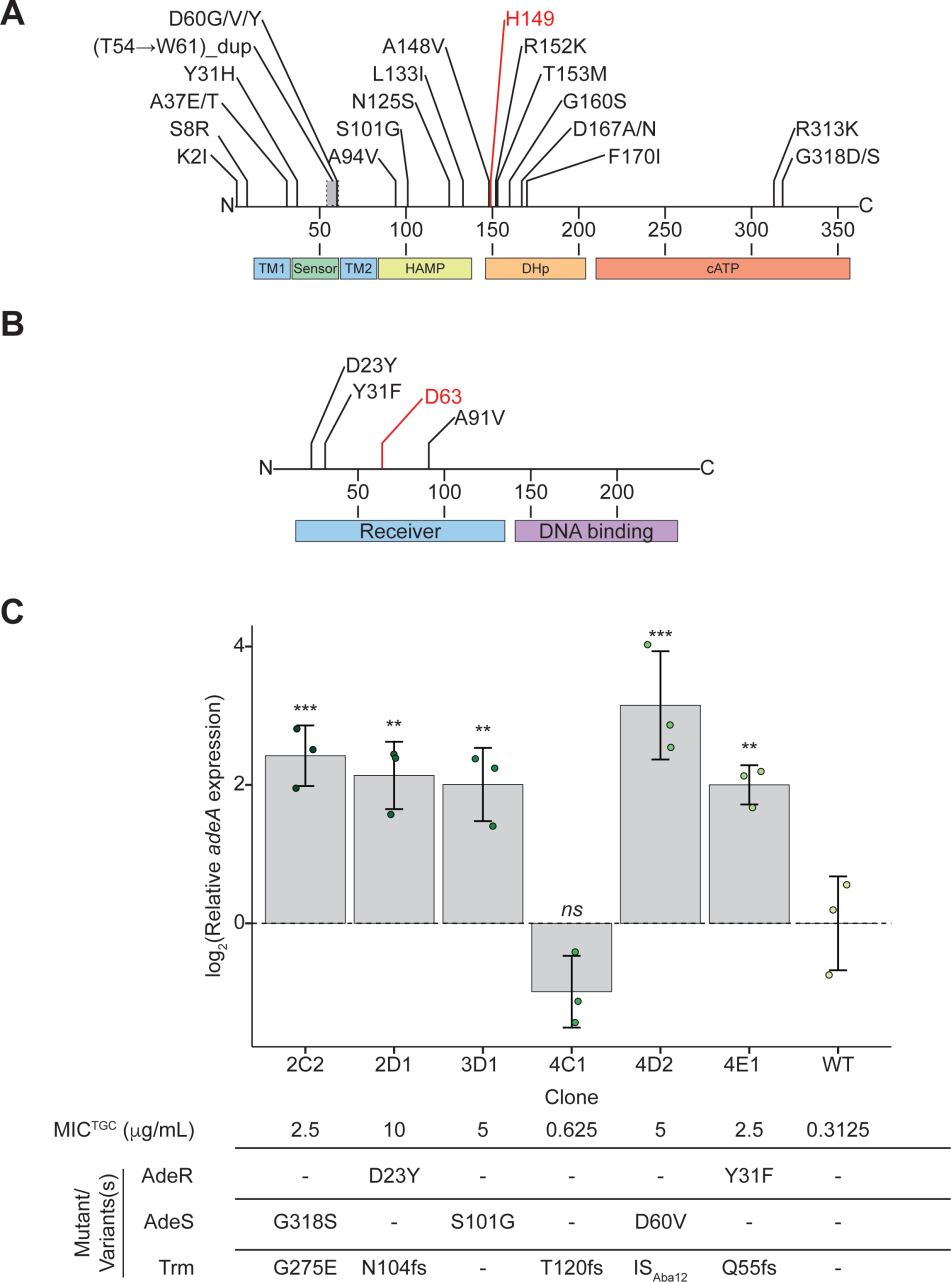
Experimentally evolved variants in AdeSR and their impact on *adeA* expression. (**A**) The distribution of variants in (**A**) AdeS and (**B**) AdeR from the two TGC evolution experiments performed with ATCC 17978 and BAA-747, with the domain features shown below the plots. For AdeR and AdeS, the phosphorylated aspartic acid and catalytic histidine, respectively, are shown in red. (**C**) Relative *adeA* expression in isolated ATCC 17978 clones containing AdeSR variants. Biological replicates are shown as points (*n* = 3), error bars represent the standard deviation, and statistical significance relative to WT is shown (*P < .05, **P < .01, ***P < .001), with the statistical analysis of significance between all comparisons available in Table S2. TGC MICs and associated genotypes are shown below the bar chart.

From the 41 instances of *adeAB*-upregulating mutational events (26 unique events; Table 1) captured across both strains during TGC resistance evolution, mutations in the histidine-kinase component of the AdeSR TCS, *adeS*, were the most prevalent resistance drivers (23 total events with 18 unique mutational positions). These variants localized to all domains within the protein, but the DHp and sensor domains were the most frequently impacted, harboring 10 and 6 mutations, respectively (Fig. 2A). Twelve *adeS* mutational variants were isolated as clones (three from ATCC 17978 and nine from BAA-747), yielding MIC^TGC^ changes of up to 32-fold. However, the contribution of each to MIC^TGC^ was challenging to delineate, given the presence of mutations in several genes in most of the clones. Three isolated ATCC 17978 mutants were used to evaluate the effect of *adeS* mutations on *adeA* expression using RT-qPCR, each originating from a different AdeS domain (ATP, HAMP, Sensor). A consistent 4.0- to 8.9-fold increase in *adeA* expression was observed across all variants, with no statistically significant differences between them (Table S2).

Aside from *adeS* mutants, we also detected *adeR* missense mutations and the insertion of IS_Aba1_ upstream of the *adeAB* operon. In *adeR*, all three mutations were localized to the N-terminal AdeS-binding domain (Fig. 2B). Like mutations in *adeS*, these *adeR* events resulted in up to 32-fold increases in MIC^TGC^ in both strains, affording comparable increases in *adeA* expression (4.0- to 4.4-fold increases, showing no statistically significant difference from the effects observed in *adeS* mutant clones; Fig. 2C; Table S2). Another *adeAB-*overexpressing event beyond *adeSR* mutations, which occurred in ATCC 17978, was the insertion of IS_Aba1_ upstream of *adeA*. Functionally, this genomic rearrangement was synonymous with an *adeS* mutation as evidenced by the same 32-fold increase of MIC^TGC^ in two isogenic clones AdeS:Gly318Ser/*trm*:IS_Aba1_/*abeM*:IS_Aba1_ and us_*adeA*:IS_Aba1_/*trm*:IS_Aba1_/*abeM*:IS_Aba1_.

Disruptive mutations (frameshifts, premature stop gains, IS element insertions, and some missense mutations) localizing in *trm* were observed in all evolving bacterial populations later than or at a similar time point to the onset of the *adeSR* mutations (Fig. 1A and B; Fig. S2A and B). These disruptions in *trm* provided a two-fold increase in MIC^TGC^ when they were isolated as single-driver events in the ATCC 17978 background but did not alter *adeAB* expression. To validate the impact of the *trm* disruption, we generated an *A. baumannii* ATCC 17978 ∆*trm* strain that exhibited the same twofold increase in MIC^TGC^. When *trm* disruptions were combined with *adeAB*-overexpressing driver mutations, consistent 16-to 32-fold increases in MIC^TGC^ were measured relative to the parental wild-type (WT) strains.

In addition to the core mutations impacting *adeAB* and *trm* genes, other genes were also mutated, albeit often at later time points, to a lesser degree within the population, and/or in a strain-specific manner. Examples of these include (i) disruptive events in *adeN*, the gene encoding the negative transcriptional regulator of the AdeIJK efflux pump in both strains; (ii) IS_Aba1_ insertions upstream of *abeM*, a Na^+^-driven multidrug efflux pump of the MATE (multidrug and toxin extrusion) family in ATCC 17978; and (iii) multiple events centering around the control of RNA transcription and stability in BAA-747 (Table 1), including *nusA* (encoding transcription termination protein NusA), *rpsJ* (SSU ribosomal protein S10p), and *rpoB/C* (DNA-directed RNA polymerase beta and beta′ subunits). The specific contribution of these variants to TGC resistance was typically impossible to delineate, given the lack of clones containing only these mutations or appropriate near-isogenic counterparts.

### Evolution of colistin resistance

Two morbidostat runs of both *A. baumannii* strains with colistin (COL) were performed using the same workflow (Fig. S1C and D) as described above for TGC. During the continuous growth of cultures of each strain in six bioreactors of the morbidostat over 94 and 99 h, respectively, the drug concentration was gradually increased to 32 mg/L (in the first 2 days) and then to 320 mg/L. The 24 samples collected from the ATCC 17978 run (four time points: 48, 57, 77, and 94 h from six reactors) and the 24 samples from the BAA-747 run (four time points: 30, 48, 79, and 96 h from six reactors) were analyzed by Illumina WGS with 500–1,000× genomic coverage. Upon identification and ranking of mutational events in potential driver genes (Table 1; Table S3A and B) by the same criteria (Supplementary Methods), we selected and characterized 23 and 12 non-redundant clones in the ATCC 17978 and BAA-747 strain backgrounds, respectively, connecting mapped mutations with MIC^COL^ values (Table S3C and D). We observed a very significant increase in MIC^COL^ values, from 0.625 µg/mL for both unevolved strains to a maximum of >160 µg/mL in selected mutant clones.

Both morbidostat runs also resulted in complex evolutionary trajectories, similarly characterized by the accumulation of multiple genomic variants, including point mutations, short indels, IS insertions, and loci copy number variations (see Table S3A and B). In multiple reactors, the combination of these variants collectively reached over 100% abundance by the end of the run (Fig. 1C and D; Fig. S2C and D), indicating the presence of double and triple mutants. The genes most prominently implicated by mutational variants are presented in Table 2.

**TABLE 2:** Major mutational variants detected during experimental evolution of COL resistance in *A. baumannii* strains ATCC 17978 and BAA-747^*c*^^,^^*d*^

Gene	ATCC 17978			BAA-747			Short functional description
	Variants	A_MAX_^*a*^	N_ALL_^*b*^	Variants	A_MAX_^*a*^	N_ALL_^*b*^	
PmrAB two-component signaling (TCS) system: regulation of PmrC-driven LPS modification							
*pmrA*	**I13N**; **L20W**; ∆L41	**98%**	**3**	I13M; L20S	14%	**2**	TCS sensor histidine kinase PmrA
*pmrB*	**∆K30; P233H; L267W;** H392Y	**98%**	**4**	**P233S; T235I; R263H;** Q265P	**98%**	**4**	TCS transcriptional activator PmrB
*pmrC*	R109C; **R109H**; D337N; D337G	**98%**	**4**				Lipid A phosphoethanolamine transferase PmrC
Lipid A biosynthesis							
*lpxA*	(S187-A193)x2	18%	**1**				Acyl-ACP-UDP-GlcNAc O-acyltransferase
*lpxC*	**V24E**	**87%**	**1**	**G87E**; G105S; S171P	**50%**	**3**	UDP-3-O-[3-hydroxymyristoyl] GlcNAc deacetylase
*lpxD*	*us_IS^f^*	39%	**1**				UDP-3-O-[3-hydroxymyristoyl]GlcN N-acyltransferase
AdeSR TCS system: transcriptional regulation of AdeAB efflux pump							
*adeS*	G160S	8%	**2**				TCS sensor histidine kinase AdeS
*adeR*	D93Y; D23V; D23Y	32%	**2**				TCS transcriptional activator AdeR
GacSA TCS system: global regulator of numerous pathways							
*gacS*				**M1?**; M203fs^*e*^; Q211^*d*^; **Q288^*d*^**; L443fs; G512fs; L552fs; E565^*d*^; L810^*d*^	**80%**	**9**	Sensor histidine kinase GacS of the TCS regulating >600 genes involved in virulence, biofilm and pili formation, motility, etc.
Other potentially significant variants							
*coaBC*				H80Y; **P156L**	**73%**	**3**	Bifunctional PPDC/PPCS in CoA biosynthesis
*mnmE*	D381fs; E380fs	37%	**3**				tRNA-5-carboxymethylaminomethyl-2-thiouridine(34) synthesis proteins MnmE and MnmG
*mnmG*	**(V11-I12)x2**	**85%**	**1**				
*clsC*	**H188fs**; **IS**	**57%**	**5**				Phosphatidyl-EtN-utilizing cardiolipin synthase
*yfiR*	**L146S**	**98%**	**1**				Transcriptional regulator of YfiRNB operon
*sdsA*	*intergenic*	**98%**	**1**				Nonaprenyl diphosphate synthase
*mlaA*	*IS*	18%	**1**				Outer membrane phospholipid-binding lipoprotein MlaA
*mlaF*	*IS*	**52%**	**1**				Phospholipid ABC transporter ATP-binding protein MlaF
*manB*	**D248-R249insGD; IS**	**73%**	**5**				Phosphomannomutase

^*a*^ A_MAX_: maximal relative abundance (%) reached by a variant in at least one sample.

^*b*^ N_ALL_: number of independent occurrences of a variant across all reactors.

^*c*^ Boldface type indicates variants reaching A_MAX_ ≥ 50%.

^*d*^ stop.

^*e*^ fs, frameshift.

^*f*^ IS, insertion of a mobile element (*us_IS*, insertion in the upstream of a respective gene).

In response to increasing COL concentrations within the reactors, these *A. baumannii* strains exhibited distinct but similar mutational profiles (Fig. 1C and D; Fig. S2C and D). The predominant drivers leading to COL resistance revolved around altering the phosphoethanolamination state of the lipid A moiety of LPS, a well-documented mechanism of COL resistance in *A. baumannii* (24, 25). In our experiments, this occurred through the acquisition of missense mutations in (i) the two-component system (TCS) *pmrAB*, which regulates the expression of the phosphoethanolamine (pEtN) transferase *pmrC*^26^, ^27^, and (ii) in the *pmrC* gene itself in combination with *pmrB* mutations (only in ATCC 17978).

In the early stage of evolution (during the first 20–30 h), bacterial populations were dominated by missense mutations in *pmrB* and, to a lesser extent, *pmrA* genes (Fig. 1C and D). We have also observed an early emergence of mutations in the *lpxC* gene (with an average Amax of ~15%) that were outcompeted and disappeared from populations after 2 to 3 days. Subsets of additional mutations emerged and accumulated by the end of evolutionary runs (80–94 h) at various levels (from 10% to 70%) were distinct and non-overlapping between the two strains. In the case of ATCC17978, the most prominent mutations were missense mutations in the genes *pmrC* and *mnmEG*, as well as disruptive SNVs and IS element insertions in the genes *manB* and *clsC*. A later stage of evolution in the BAA-747 strain was dominated by the emergence and accumulation of disruptive SNVs and IS element insertions in the gene *gacS* (reaching an average Amax of ~70%) within the population entirely dominated by *pmrB* mutational variants (with an aggregated average Amax of ~97%).

Missense mutations in the *pmrB* gene encoding the sensor histidine kinase of the PmrAB TCS were the most frequent events observed in 10 out of 12 reactors (four in ATCC 17978 and six in BAA-747; Table 2). Of the total 16 detected variants, two variants (PmrB:Lys30del, PmrB:His392Tyr; Amax ≥ 10%) were identified for the first time. Among the isolated clones, 13 of the potential 16 *pmrCAB* variants were represented, most commonly in combination with other (non-*pmrCAB*) variants (Table S3C and D).

**Fig 3:**
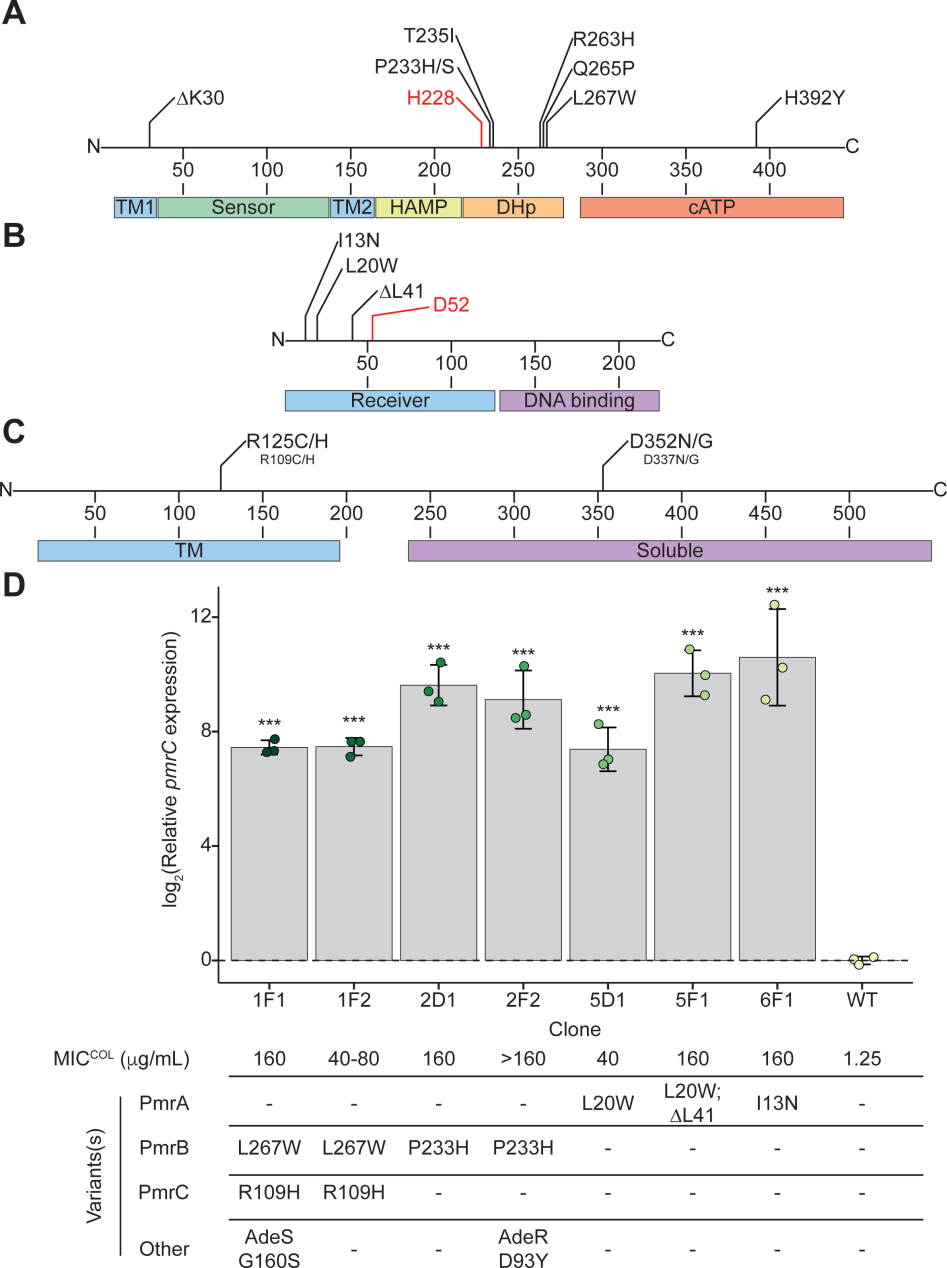
Experimentally evolved variants in PmrABC and their impact on *pmrC* expression. The distribution of variants in (**A**) PmrB, (**B**) PmrA, and (**C**) PmrC from the two COL evolution experiments performed with ATCC 17978 and BAA-747, with the domain features shown below the plots. For PmrA and PmrB, the phosphorylated aspartic acid and catalytic histidine, respectively, are shown in red. (**D**) Relative *pmrC* expression in isolated ATCC 17978 clones containing PmrABC variants. Biological replicates are shown as points (*n* = 3), error bars represent the standard deviation, and statistical significance relative to WT is shown (*P < .05, **P < .01, ***P < .001), with the statistical analysis of significance between all comparisons available in Table S4. COL MICs and associated genotypes are shown below the bar chart. All *pmrAB* mutations elicited *pmrC* overexpression, with statistically insignificant differences in the magnitude of the effects between distinct PmrB variants. Conversely, different *pmrA* mutations induced different levels of *pmrC* overexpression, with the PmrA:Leu20Trp clone invoking a significantly lower expression than other isolated clones—an impact reflected by its twofold reduced MIC^COL^ relative to other clones.

In five out of seven non-redundant PmrB variants, amino acid substitutions were localized to the dimerization histidine phosphotransfer domain (DHp) near the catalytic His228 (Fig. 3A). The most prominent PmrB variants, such as PmrB:Pro233Ser and PmrB:Thr235Ile in the BAA-747 strain, increased MIC^COL^ by approximately 64-fold (Table S3C and D). In the ATCC 17978 strain, PmrB:Pro233His and PmrB:Leu267Trp upregulated *pmrC* expression by 169- to 776-fold and increased MIC^COL^ up to 256-fold (Table S3C, D and S4).

Five variants spanning three unique residues across the two strains were localized within the N-terminal receiver domain (PmrB binding domain) of the response regulator PmrA, the second component of this TCS (Fig. 3B). Three of these events (PmrA:Leu20Trp/Ser and PmrA:Leu41del) had not previously been reported. In the ATCC 17978 background, the impact of isolated PmrA variants on both *pmrC* expression and MIC^COL^ was comparable to that of PmrB variants, with increases of 169- to 1,552-fold and 256-fold, respectively (Fig. 3D; Table S4).

Mutations in *pmrC* were less prevalent than in *pmrAB* and always occurred alongside *pmrB* mutations, precluding an independent assessment of their contributions to COL resistance. Variants PmrC:Arg109Cys/His, previously reported as an arginine to proline substitution (28), localized to the transmembrane domain at the interface between the outer membrane and extracellular region, while variants PmrC:Asp337Asn/Gly localized to the soluble domain (Fig. 3C).

Nearly all isolated clones harbored additional mutational events occurring either before or after *pmrCAB* mutations. The most ubiquitous of these were IS insertions and disruptive mutations in the *gacS* gene encoding the signaling kinase GacS that occurred only in the BAA-747 strain and combined with *pmrB* mutations at later time points. Mutations in *gacS* were observed in most isolated clones and always in combination with PmrB variants, seemingly yielding a ~4-fold additional increase in MIC^COL^. Three other types of mutational events were captured in BAA-747 (Table 2): (i) three missense variants in *lpxC*, encoding a UDP-3-O-[3-hydroxymyristoyl] *N*-acetylglucosamine deacetylase involved in LPS biosynthesis (29) (LpxC:Gly87Glu, LpxC:Gly105Ser, and LpxC:Ser171Pro), which occurred at early stages and were outcompeted by *pmrB* mutations due to their insignificant impacts on MIC^COL^ (Table S3D); (ii) two missense variants in *coaBC*, encoding a bifunctional enzyme phosphopantothenoylcysteine decarboxylase (EC 4.1.1.36)/ phosphopantothenoylcysteine synthetase (EC 6.3.2.5) in coenzyme A biosynthesis (CoaBC:His80Tyr and CoaBC:Pro156Leu) that occurred cumulatively in three reactors at mid-late time points co-occurring with *pmrB* variants; and (iii) one variant in *rpsE* (RpsE:Ala58Tyr) encoding the SSU ribosomal protein S5p, which occurred early in one reactor but was later outcompeted by a *pmrB* mutant. The isolated LpxC:Asn90Ser (not reported in the population table as it occurred with an abundance below the threshold) and CoaBC:Pro156Leu variants in the BAA-747 background contributed to a ~4-fold increase in MIC^COL^ (Table S3D).

These mutational events did not occur in ATCC 17978, where modifications in four other pathways were mapped as secondary events, tentatively enhancing COL resistance or compensatory fitness (Table 2). One pathway involved prominent missense mutational variants in two functionally related genes, *mnmE* and *mnmG*, which encode the tRNA-5-carboxymethylaminomethyl-2-thiouridine(34) synthesis proteins MnmE and MnmG. Another pathway featured disruptive events (IS element insertion or frameshifting mutation) in *clsC* (cardiolipin synthase, which uses phosphatidylethanolamine and phosphatidylglycerol [30] and is also referred to as *pld2* [31]), observed in four reactors. For the third secondary event, *manB*, a phosphoglucomutase (EC 5.4.2.2)/phosphomannomutase (EC 5.4.2.8), was disrupted via IS element insertion or mutated through the insertion of six bases resulting in the ManB:Asp248_Arg249insGlyAsp variant. Finally, an additional pathway included missense mutations in the *adeS* and *adeR* genes observed in one reactor reaching a cumulative abundance of 100%. These mutations resulted in the upregulation of the *adeAB* operon (as discussed before and illustrated in Table S2) but did not impact *pmrC* expression (Table S4). The contributions to COL resistance could not be determined for any of these secondary mutations due to a lack of single-mutant clones or appropriate isogenic counterparts for comparison.

Diverging from the predominant evolutionary trajectory shared between both strains, *A. baumannii* ATCC 17978 in one reactor evolved COL resistance in a *pmrCAB*-independent manner (Table S3A). In this reactor, missense mutations in *lpxC* (LpxC:Val24Glu) conferred initial resistance but were quickly outcompeted by IS element insertions upstream of and inside the *lpxD* gene. These *lpxD*:IS insertion events included direct disruption of *lpxD* (17.8% A_max_) or insertion of an IS element at the 3′ end of *ompH* (38.9% A_max_), which overlaps with the σ^70^ promoter immediately upstream of *lpxD* (Table S3), likely disrupting this promoter site and subsequently downregulating *lpxD* expression. Additional mutations were also observed alongside these during this evolutionary trajectory (Table S3A).

Initial attempts to grow the population containing the *lpxD* disruptions on LB agar plates for clone isolation were unsuccessful; however, this was remedied by the addition of COL at concentrations above 5 µg/mL, which resulted in a slow but robust growth. From this population, two non-redundant clones were isolated, each harboring an IS element insertion upstream of *lpxD* (3′ end of *ompH*), mutations in *yfiR*, and mutations in the intergenic region between *sdsA* and *rplU*. Additionally, one of the clones contained an IS element insertion in *cph2_5* (diguanylate cyclase/phosphodiesterase) and *mlaA* (a lipoprotein involved in the removal of mislocalized phospholipids from the outer membrane), while the other contained a disruptive frameshift mutation in *mlaA* (MlaA:Gly273fs). The COL-dependence phenotype was retained in these clones (4F1 and 4F2 in Table S3C), and a MIC^COL^ of >160 µg/mL was measured.

### Tigecycline resistance predisposition in colistin-resistant isolates

Because a subset of COL^R^-evolved clones acquired mutations in *adeSR*, we performed a subsequent TGC evolution run in the morbidostat for these COL^R^ clones. We hypothesized that the samples containing these *adeSR* mutations would acquire more robust TGC^R^ faster than the parental samples, since only a mutation in *trm* would be required, rather than mutations in both *trm* and *adeSR*.

Using our morbidostat-based continuous culturing approach, we compared the dynamics of TGC resistance acquisition in three nearly isogenic clones selected during experimental evolution of CLS resistance in *A. baumannii* ATCC 17978: one harboring wild-type *adeSR* (PmrB:Pro233His) and two with *adeSR* mutational variants (PmrB:Pro233His/AdeS:Gly160Ser and PmrB:Pro233His/AdeR:Asp93Tyr). By performing a single morbidostat run with two reactors per each of the three clones, we observed clear distinctions between the growth behavior of *adeSR*-WT and *adeSR*-mutant clones. A robust resistance to TGC emerged quickly in the four reactors containing *adeSR*-mutant clones, while the evolution of resistance was delayed by 15 h in the reactors inoculated with *adeSR-*WT clones. After five days of evolution, all reactors, regardless of *adeSR* genotype, reached a comparable TGC resistance.

Reactors inoculated with *adeSR-*mutant clones rapidly acquired disruptive mutations or IS element insertions in *trm* during the initial time points (Table S5; Fig. S2E). In contrast, reactors with *adeSR*-WT clone first acquired *adeS* mutations (AdeS:Ile157Leu or AdeS:∆Tyr31del) before any disruptive events in *trm* were observed. Once the populations in all reactors acquired both *adeSR* single nucleotide variants (SNVs) and *trm* disruptive events, tertiary events began to occur, including disruptions in *adeN* (IS insertion, frameshift, or premature stop gain) reaching up to 100% prevalence in all reactors (except reactor 6, which reached ~41% Amax) and/or mutations upstream of *abeM* (Table S5). Notably, despite the absence of colistin in the medium, mutations in *pmrB* (PmrB:Pro233His) persisted in the evolving populations over the five-day run.

### Susceptibility testing of TGC^R^ and COL^R^ clones to other antimicrobial agents

To assess whether major TGC and COL resistance-driving mutations contribute to broad antimicrobial phenotypes, we have performed broth microdilution measurements of MIC values for representative TGC^R^ and COL^R^ clones across a panel of nine additional clinically relevant antimicrobials. The results of these measurements (Table S14) reveal a modest (2- to 4-fold) but consistent increase of MIC in AdeSR mutational variants (TGC-evolved) for some antibiotics, including meropenem, ciprofloxacin, levofloxacin, gentamicin, and minocycline (for both *A. baumannii* strain backgrounds), and additionally for tobramycin (only for BAA747 strain).

### 3D modeling and genomic distribution of tigecycline and colistin resistance-driving mutations

We used comparative genomics to generalize observations made via the morbidostat-based experimental evolution of antibiotic resistance in the context of clinical prevalence. To this end, we aimed to infer the prevalence of TGC- and COL-resistant *A. baumannii isolates* with publicly available genomes, using the data acquired in this study as a training set of known (experimentally grounded) resistance-driving mutations. One of the challenges when predicting antibiotic resistance from mutations within a gene is distinguishing neutral mutations (those that do not confer resistance) from driving mutations (those that do confer resistance). This is often the case for clinical isolates that are reported with many mutations (variations) within a single gene, while only one (or two) of them is likely to impart any resistance. This is in contrast with experimental evolution studies where the parental sequence of any gene is known, which typically allows us to circumvent this ambiguity.

While the pangenomic frequency of the variants detected in this study was our primary interest, we also sought to compare it to the distribution of all variants in these proteins within the available genome sequences. To this end, we aligned sequences of the respective TCS proteins extracted from ~10,000 high-quality *A. baumannii* genomes (from the BV-BRC collection [22]) and calculated the ungapped Shannon entropy (23) for each position in each of the four proteins of interest. We hypothesized that most genuine resistance-driving variants would be relatively rare (low Shannon entropy), while the amino acid positions with relatively high Shannon entropy values (>0.2) would largely comprise neutral variations.

**Fig 4:**
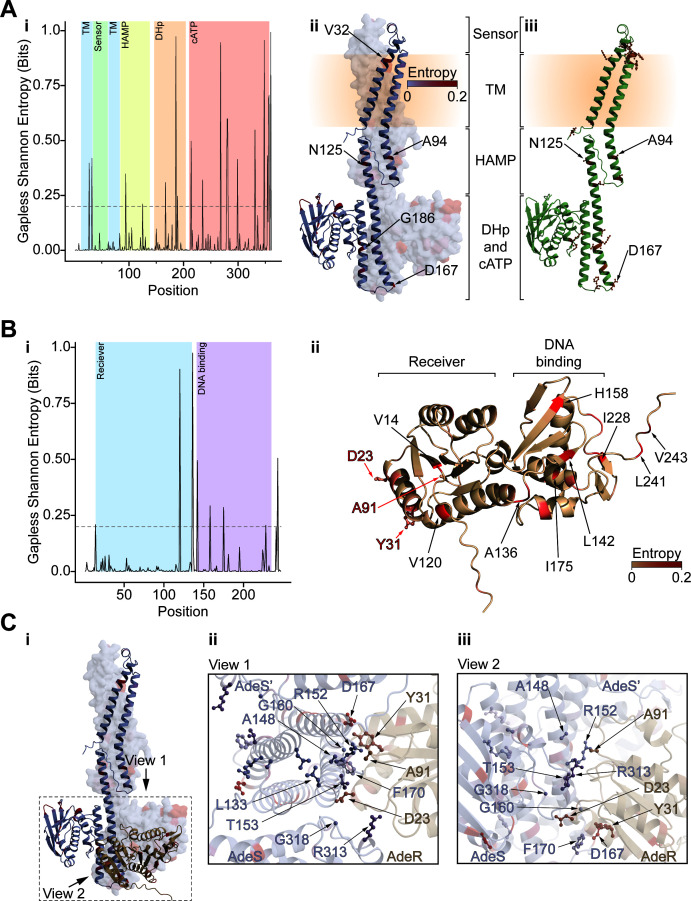
Analysis of AdeSR positional entropy and morbidostat variant distributions. (**A**) Gapless Shannon entropy shown as a (i) plot with the domains of AdeS colored or (ii and iii) shown on two angles of the structure of AdeS with the blue to red color gradient reflecting the value. In (ii), the indicated positions are those not in the catalytic ATP binding domain with entropy greater than 0.2, exceeding the threshold shown in (i). In (iii), the experimentally determined morbidostat point variants are depicted in ball-and-stick representation, and those with entropy > 0.2 are labeled. (**B**) Gapless Shannon entropy shown as a (i) plot with the domains of AdeR colored, or (ii) shown on the structure of AdeR with the brown to red color gradient reflecting the value. Morbidostat point variants are depicted in ball-and-stick representation. (**C**) (i) Model of interaction between AdeS and AdeR, with the region of interest highlighted by a dashed box and the two cutout views for (i) and (ii) being indicated by arrows. (ii) The top-down and (iii) side-on views of the AdeS-R interface, AdeS, and AdeR are colored by the gradients in (**A**) and (**B**), respectively. Morbidostat variants are depicted as ball-and-sticks and annotated.

Within the AdeSR TCS, many highly variable positions were identified in the sensor kinase AdeS and the response regulator AdeR. In AdeS, 14 of the 21 high-entropy positions were localized to the catalytic ATP binding domain (Fig. 4A; Table S6). However, some were also present in other domains, including the DHp (dimerization histidine phosphotransfer) domain (3 of 21), though not at the surface where AdeR is expected to dock based on homology modeling (Fig. 4A). Only three high-entropy positions in AdeS (Ala94, Asn125, and Asp167) were identified as antibiotic resistance drivers out of the total 17 unique variant driver positions mapped in our study. Additionally, our data for AdeS showed that driver positions were rarely found in the catalytic ATP binding domain (Fig. 4A); when they were, they occurred in the flexible ATP “lid” located to the DHp domain, unlike the high-entropy positions, which were frequently found in this domain (Fig. 4A). Most of the identified driver positions were present in the sensor, HAMP, and DHp domains—especially at the putative interfacial residues where AdeR was modeled to bind (Fig. 4C). For AdeR, both the AdeS-binding and the DNA-binding domains exhibited a comparable number of high-entropy positions (three and six, respectively; Fig. 4B), although none overlapped with residues potentially involved in either DNA or AdeS binding, with Asp63 (which AdeS phosphorylates) (32), or with the experimentally observed resistance-driving variants, which localized only to the N-terminal AdeS-binding domain at the putative interface between the response regulator and sensor histidine kinase (Fig. 4B and C).

A curated set of 30 total variants across 21 unique positions (17 variant positions from this work and additional variants isolated in prior morbidostat studies [12, 17–19]) was used as a low estimate for the ubiquity of TGC resistance in the publicly available *A. baumannii* genomes. Cumulatively, 13.7% of the assessed genomes (containing 12,541 AdeS sequences) possessed an AdeS variant identified in this study and, therefore, were interpreted as potentially TGC resistance-conferring sequences (Table S7A through C). Among them, the top three positions correspond to Ala94Thr/Val, Asn125Ser, and Asp167Ala/Asn (5.8%, 0.9%, and 5.2%, respectively), as expected due to their high positional entropy. Outside of these positions, the abundance of our captured variants ranged from 0 to 0.3%, totalling 1.8%.

Performing the same analysis for the three AdeR variant positions (four total variants) yielded a similar picture: 1.1% of sequences being annotated as resistant, 11.7% as susceptible, and 87.2% as unknown (due to the presence of uncharacterized variants) out of a total of 13,191 AdeR sequences with the abundance of resistance-driving variants at a given position ranging from 0 to 0.9% prevalence (for a total of 1.1%) (Table S8A through C).

**Fig 5:**
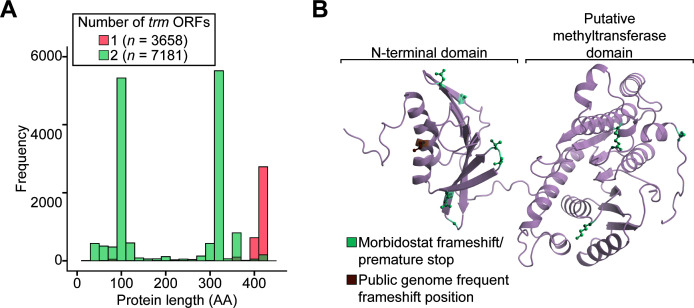
Disruptions in the *trm* ORF across public genomes and in experimental evolution. (**A**) The frequency of *trm* ORF disruptions across public *A. baumannii* genomes is indicating the prevalence of genomes with two (green) *trm* ORFs occurring at a relatively well-conserved position. (**B**) The distribution of experimental *trm* premature stop mutations and frameshifts (green) and well-conserved public frameshifts (orange) projected onto the structure of Trm.

Outside of *adeSR* mutations central to TGC resistance, the methyltransferase *trm* was consistently disrupted during TGC^R^ evolution and was, therefore, an additional mutational event that we examined in publicly deposited genomes. Analysis of 10,839 *A. baumannii* genomes containing *trm* encoding sequences revealed that 7,181 (approximately 66%) contained a frameshifted version of *trm* (Fig. 5A; Table S9A and B). Not only was the presence of this disrupted form ubiquitous, but the corresponding frameshift also occurred at a highly conserved position with 5,208 genomes (72.5% of those with the disruption, 48% of all genomes analyzed) encoding separate 91 amino acid N-terminal and ~320 amino acid C-terminal ORF segments. This frameshift occurred in the middle of the predicted ⍺2 helix in the N-terminal domain. The C-terminal methyltransferase domain remained unaffected (Fig. 5B). From the morbidostat-based TGC^R^ evolution in the two *A. baumannii* strains, we observed a similar localization of acquired frameshift mutations in *trm*, with six out of the nine frameshift or premature stop mutations being at the 5′ end of the gene (Fig. 5B), resulting in disruption either within the N-terminal domain or the linker between the N- and C-terminal domains.

Due to the accumulation of dual *adeSR/trm* mutational variants during TGC^R^ evolution, we also examined the prevalence of sequenced genomes with both *trm* frameshift disruptions and resistance-driving *adeSR* mutations in the same set of public genomes. Of the 1,860 genomes that contained an AdeS and/or AdeR resistance-driving variant, 558 (or ~30%) contained a frame-shifted version of *trm* and, thus, might be assumed to manifest a full-scale TGC resistance (Table S10).

**Fig 6:**
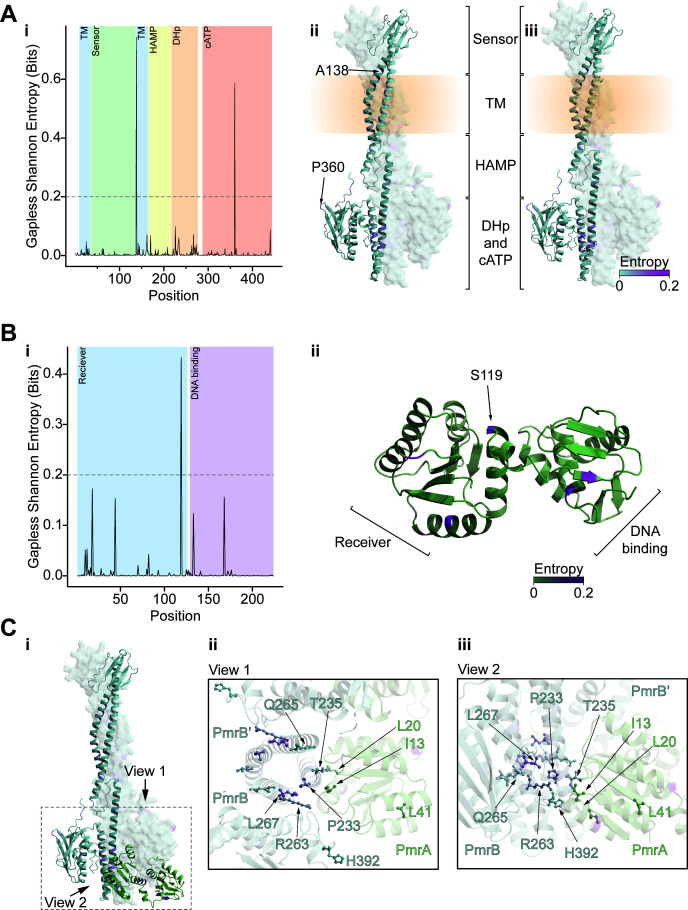
Analysis of PmrAB positional entropy and morbidostat variant distributions. (**A**) Gapless Shannon entropy shown as a (i) plot with the domains of PmrB colored or (ii and iii) shown on two angles of the structure of PmrB with the cyan to magenta color gradient reflecting the value. In (ii), the indicated positions are those not in the catalytic ATP binding domain with entropy greater than 0.2, exceeding the threshold shown in (i). In (iii), the experimentally determined morbidostat point variants are depicted in ball-and-stick representation, and those with entropy >0.2 are labeled (if any). (**B**) Gapless Shannon entropy shown as a (i) plot with the domains of PmrA colored or (ii) shown on the structure of PmrA with the green to magenta color gradient reflecting the value. Morbidostat point variants are depicted in ball-and-stick representation. (**C**) (i) Model of interaction between PmrB and PmrA, with the region of interest highlighted by a dashed box and the two cutout views for (i) and (ii) being indicated by arrows. (ii) The top-down and (iii) side-on views of the PmrA-B interface, PmrB, and PmrA are colored by the gradients in (**A**) and (**B**), respectively. Morbidostat variants are depicted as ball-and-sticks and annotated.

Unlike in the case of AdeSR TCS, there were very few instances of high-entropy positions in either component of PmrAB, with no positions overlapping with those identified as resistance-driving from our experiments (Fig. 5A and B; Tables S11A through C and S12A through C). In PmrB, the high-entropy positions were localized to the interface between the sensor and transmembrane (TM) domains (Ala138), as well as the catalytic ATP binding domain (Pro360) (Fig. 6A; Table S11A through C). The analysis of the prevalence of our experimentally isolated PmrB variants demonstrated that they were infrequently captured in previously sequenced genomes ranging from 0 to 0.43% abundance with a total frequency of 0.7%. For all the remaining sequences, 54.3% were classified as susceptible and 44.9% as unknown due to similar constraints as outlined above. In the response regulator PmrA, the only high-entropy position (Ser119) localized to the end of ⍺5 of the N-terminal PmrB binding domain, close to the linker between this domain and the C-terminal DNA-binding domain (Fig. 6C). For PmrA, there was only one instance of a resistance-driving variant detected in the public genomes (<0.1% prevalence), leaving 85.7% susceptible sequences and 14.3% with unclassified variants across all public sequences (Table S12A through C).

## DISCUSSION

Tigecycline (TGC) and colistin (COL) are both considered last-resort antibiotics for treating drug-resistant *Acinetobacter baumannii* infections (33, 34), and proper stewardship of their utilization is essential to prevent widespread resistance from developing (35). Understanding antibiotic resistance mechanisms is a cornerstone in establishing appropriate usage guidelines for specific classes of antibiotics. Here, we demonstrated that the experimental evolution of resistance toward each of these classes of antibiotics predominantly relied on mutations in two separate two-component systems (TCSs), *adeSR* and *pmrAB*, regulating the expression of the *adeAB(C)* efflux pump and the lipid-A phosphoethanolamine transferase, *pmrC*, respectively. We identified numerous unique point mutations in genes from these TCSs that resulted in overexpression of the corresponding operons and enhanced MICs, in line with prior studies (24, 25, 36–39).

Overexpression of the *adeABC* efflux pump (denoted *adeAB(C)* herein due to the lack of *adeC* in many strains of *A. baumannii,* including both ATCC 17978 and BAA-747), driven by various AdeSR variants, is a well-established mechanism of TGC resistance, with numerous variants identified in clinical isolates (40–43). Our study builds on these findings, broadening the spectrum of resistance-driving mutations observed in *A. baumannii*.

AdeS variants mapped in this study mainly localized to the sensor, HAMP, and histidine kinase domains, emphasizing the range of potential positions, whereby resistance-driving mutations can occur within this histidine kinase. In AdeR, all detected variants localized to the N-terminal receiver domain, which is expected to interact with AdeS. The high frequency of *adeS* and *adeR* mutations, which resulted in interfacial variants within the AdeSR complex, likely reflects the importance of these interactions for controlling the degree of kinase activity (44, 45), the specificity of this activity (46), and the lifetime of the resultant phosphorylated response regulator (47). In other TCSs, mutations in both the sensor and HAMP domains have also been reported to impact the activity of the kinase component through alterations in the intradimer interactions (48) or allosteric modulation of the signal transduction (49). Although a rare event in our study, mutations in the catalytic ATP binding domain are able to impact the activity of the AdeS, likely affecting the kinase/phosphatase ratio in a similar manner to that which was reported for PhoQ (50). From the plethora of potential mechanisms through which TCS mutations may impact the expression of their regulated operon, it has been reported that in the case of AdeSR variants, AdeR likely impacts the stability of the protein, while mutations in specific regions of AdeS (namely the HAMP domain) prevent the dephosphorylation of AdeR, both of which have the potential to increase the expression of the *adeABC* operon (51). Regardless of the specific mutation and its potential mechanistic impact, variants localizing to either component of the AdeSR TCS elicited a similar level of *adeAB operon* overexpression relative to the parental WT strain. This suggests that variants captured in this system likely function in a similar manner—by increasing the expression of genes under the control of the AdeSR system. Compared to the *adeSR* variants reported in the literature (Table S13), our results revealed a remarkable degree of domain specificity and relatively rare prevalence in public genomes.

While the development of TGC resistance in *A. baumannii* is often attributed primarily to efflux pump overexpression, other mechanisms also enhance tolerance to the drug (52). One such mechanism involves the disruption of the putative methyltransferase *trm*, which has been recorded in TGC-resistant clinical isolates (53, 54) and frequently emerged in our experiments. The exact function of *trm* and the advantages of its disruption remain unclear. However, our genome mining observation that ~2/3 of publicly available *A. baumannii* genomes contained *trm* with a frameshift mutation suggests that these strains may already possess a predisposition to TGC resistance. Our experimental findings support the hypothesis that *trm* frameshifting mutations are functionally synonymous with complete gene loss, as an engineered ATCC 17978 Δ*trm* strain exhibited the same 2× increased MIC^TGC^ as our selected clones 1E2 (*trm*:ISAba11) and 4C1 (*trm*:Thr120fs). Taken together, these findings suggest that *trm* disruption is a key step in TGC resistance evolution, which preexists in many *A. baumannii* isolates.

The evolution of COL resistance in *A. baumannii* was marked by the prevalence of point mutations in the *pmrCAB* locus, consistent with literature linking pEtN modification of LPS to COL^R^ phenotype (24, 25, 39). Specific missense mutations in *pmrAB* are known to upregulate *pmrC*, the enzyme responsible for lipid-A pEtN decoration, which prevents COL-LPS interactions and is a well-established mechanism of COL resistance (55). While PmrAB TCS also regulates other operons, including the *naxD*-containing operon involved in the transfer of GalN to LPS, their contributions to COL resistance are considered less significant than PmrC-driven modification, as demonstrated by studies using a series of *A. baumannii pmrC* and *naxD* deletion mutants (56).

Many *pmrA* and *pmrB* mutations were found in interfacial regions of the PmrAB complex, particularly in the N-terminal domain of PmrA and the DHp domain of PmrB, similar to what was observed in AdeSR-driven TGC resistance described above. These variants may enhance TCS activity by altering the interactions between the components of the complex (57), or through other impacts of mutations in these proteins (as discussed above for AdeSR TCS). Ultimately, all variants resulted in considerable *pmrC* overexpression. While most PmrB variants localized to the DHp domain, a deletion in the transmembrane domain (PmrB:Lys30del) close to the Ala28Val mutation known to enhance *pmrC* expression (28) and a mutation in the ATP-lid of the ATP binding domain suggest that PmrB activity can be enhanced through mechanisms beyond interfacial alterations. This aligns with other studies identifying PmrB variants outside the DHp domain (58). In addition to the alteration of the regulation of LPS-pEtN modifications through *pmrC* overexpression, mutations in *pmrC*—occurring only alongside *pmrAB* mutations—may also have contributed to COL resistance in some *A. baumannii* clones. This phenomenon has been previously reported, with mutations in the transmembrane domain of PmrC (PmrC:Ile42Val and PmrC:Leu150Phe) (59). Similarly, the PmrC:Arg109His variant, which also localized to the N-terminal TM domain, observed in our study, was previously reported in combination with PmrA:Gly54Glu and was shown to confer COL resistance (60).

COL resistance outside of *pmrCAB* operon in *A. baumannii* primarily arises from suppression of LPS biogenesis through *lpxA*/*C*/*D* mutations and IS element disruptions, both *in vitro* (61, 62) and in clinical isolates (61, 63), though with reduced virulence in mouse infection models (64). While subpopulations harboring *lpxC* missense mutations were observed in both strains during our COL evolution experiments, they were not sustained as they did not confer robust resistance. Instead, LPS loss likely contributed to COL resistance in populations that possessed a combination of IS element insertions upstream or within *lpxD*, along with disruptions in *mlaA*. Although we did not directly test for LPS loss in these clones, the disruption of LPS synthesis was anticipated in these populations, as *mlaA* disruption has been reported as a compensatory mechanism for overcoming growth deficiencies associated with LPS loss (65–67). These clones exhibited unusual growth behavior, growing only on media supplemented with COL, a phenomenon that has been previously observed in LPS-deficient *A. baumannii* strains (68, 69), putatively caused by the increased phosphatidylglycerol content of the outer membrane and subsequent bilayer strengthening caused by interactions with COL (70, 71).

Our observation of a subset of clones with mutations in either *adeS* or *adeR*, in addition to *pmrB* mutations, suggests a potential role for *adeSR* mutations—and consequently *adeAB* upregulation—in COL resistance. Previous studies have implicated efflux pumps, including AdeABC, in COL resistance through observations, such as upregulation in resistant isolates (72, 73) and the increase of COL sensitivity with efflux pump inhibitors (73), with similar findings noted for other polymyxins (74). However, clones that evolved in the presence of TGC and acquired *adeSR* mutations did not exhibit elevated MIC^COL^, consistent with earlier studies (75, 76). This suggests that the contribution of the AdeAB(C) efflux pump may differ in pEtN-modified LPS-harboring cells compared to those with native LPS. Aside from their potential role in COL resistance, the evolution of *adeSR* mutations demonstrated that developing COL resistance during treatment may trigger a predisposition to TGC resistance.

Outside of the decreased susceptibility of these experimentally evolved TGC^R^ and COL^R^ clones towards TGC and/or COL, we observed a general decrease in susceptibility for TGC-evolved clones to gentamicin, minocycline, and ciprofloxacin, among other compounds. This was consistent with the expected impact of efflux upregulation in these clones (17, 77, 78). Notably, clones harboring a disruptive mutation in *trm* exhibited increased minocycline MICs, suggesting that the mechanistic contribution of this mutation, although not yet defined, likely confers resistance across the tetracycline class of antibiotics. No such effects were observed for the COL-evolved clones featuring PmrAB mutational variants, as no impact on efflux was noted in these clones.

Overall, our morbidostat-based studies on experimental evolution of resistance to two last resort antimicrobials, tigecycline and colistin, in *A. baumannii* recapitulated and extended the existing knowledge of the two resistance mechanisms driven by a subset of missense mutations in two TCSs, AdeSR and PmrAB, respectively. The clinical relevance of resistance-driving variants identified in morbidostat-based studies is underscored by the observation that most mutations—and their combinations—do not significantly affect bacterial fitness. Growth curve analyses show no appreciable changes in doubling time for nearly all representative drug-resistant clones from the four morbidostat experiments compared to the unevolved parental strains (Fig. S4A thorugh C). This is likely due to a key feature of morbidostat technology: continuous dilution of cultures, which imposes negative selection against slow-growing mutants. This dynamic more closely mimics the progression of infections in a clinical setting.

The obtained compendium of experimentally grounded driving mutations, combined with the analysis of positional variations in the respective genes over a large collection of *A. baumannii* genomes, paves the way for predictive resistomics with translational implications in antibiotic stewardship.

We have assessed the distribution of AdeSR- and PmrAB-driven TGC- and COL-resistant variants across both geography and MLST by leveraging these types of metadata captured in the BV-BRC database. The results of this analysis are presented in the form of comparative tables and graphs in Table S7D, S8D, E, S11D, S12D and E. Despite some caveats (e.g*.*, that a substantial fraction of genomes, up to 85% for AdeRS-variants, contain variants with yet *unknown* implications for drug resistance/susceptibility), this analysis shows quite an uneven distribution of definitively mapped resistant variants, both by geography and MLST. Thus, for AdeRS-driven resistance, four countries featured from 53% to 81% of isolate genomes with predicted TGC resistance, while 11 countries displayed 0% predicted TGC-resistant AdeRS variants. Additionally, 32 countries (including the largest sets of genomes from the USA and China) presented resistance frequencies ranging from 2% (China) to 20% (USA). It is tempting to speculate that such a diversity may at least in part reflect local trends in prescription of TGC-type antibiotics. An even more contrasted picture was observed for MLST distribution, with 10 large ST clusters (>30 genomes per cluster) presenting 36%–100% and 15 clusters featuring 0% of AdeRS-driven resistant variants. The largest MLST cluster 2.2 (containing 4,685 *A. baumannii* genomes according to BV-BRC classification schema) had a very low frequency of predicted resistance (~1%). The overall frequency of PmrAB-driven COL resistance (0.91%), which comprised nearly exclusively of PmrB variants, was too low to assert any significant trends in geographic or MLST distribution.

When considered alongside our previously published studies (12, 17–19) on other antimicrobials across various bacterial species, this study highlights the potential of the morbidostat-based experimental evolution workflow as a powerful tool for mapping and understanding the resistome of diverse bacterial pathogens, including members of the ESKAPE group.

## Data Availability

Clonal and population sequencing data are available in the SRA database under BioProject accession number PRJNA1232934 (aside from ‘A’ samples corresponding to the starting points for *A. baumannii* ATCC 17978, which were described previously [17] and are available in another BioProject: reactor 1 - BioSample: SAMN37382541, reactor 2 - BioSample: SAMN37382545, reactor 3 - BioSample: SAMN37382549, reactor 4 - BioSample: SAMN37382553, reactor 5 - BioSample: SAMN37382557, reactor 6 - BioSample: SAMN37382561). The *A. baumannii* ATCC 17978 reference genome is available in the European Nucleotide Archive under sample accession number ERS4228590.

## SUPPLEMENTAL MATERIAL

Supplemental material aac.00809-25-s0001.docx Supplemental methods; Fig. S1 to S4.

Supplemental tables aac.00809-25-s0002.xlsx Tables S1 to S14.

ASM does not own the copyrights to Supplemental Material that may be linked to, or accessed through, an article. The authors have granted ASM a non-exclusive, world-wide license to publish the Supplemental Material files. Please contact the corresponding author directly for reuse.
