# Supplementary material for: Mutations in two-component signaling systems drive experimental evolution of tigecycline and colistin resistance in Acinetobacter baumannii
Source: Antimicrob Agents Chemother. 2025 Oct 29;69(12):e00809-25. doi: 10.1128/aac.00809-25 (PMC12691633; doi:10.1128/aac.00809-25)
Supplement: Supplemental material — Supplemental methods; Fig. S1 to S4. [file aac.00809-25-s0001.docx]

**Mutations in Two-component Signaling Systems Drive Experimental Evolution of Tigecycline and Colistin resistance in *Acinetobacter baumannii***

Kent J.E.^1^, Elane M.^1^, Leyn S.^1^, Zlamal J.^1^, Wong N.^1^, Aizin M.^1^, Trauner A. ^2^, Zampaloni C.^2^, Louvel S.^2^, Haldimann A.^2^, Vercruysse M.^2^, Osterman A.^1,*^

^1^ Sanford Burnham Prebys Medical Discovery Institute, La Jolla, California, USA

^2^ Roche Pharma Research and Early Development, Infectious Diseases Therapeutic Area, Roche Innovation Center Basel, F. Hoffmann-La Roche, Basel, Switzerland

*Corresponding author: [osterman@sbpdiscovery.org](mailto:osterman@sbpdiscovery.org)

**Table of Content**

[I. Supplementary Figures 3](#_Toc202780270)

[Supplementary Figure S1. 3](#_Toc202780271)

[Supplementary Figure S2. 7](#_Toc202780272)

[Supplementary Figure S3 13](#_Toc202780273)

[Supplementary Figure S4. 14](#_Toc202780274)

[II. Supplementary Methods 15](#_Toc202780275)

[Morbidostat setup and programming 15](#_Toc202780276)

[Sequencing data analysis, variant calling and ranking 15](#_Toc202780277)

[Population WGS data deconvolution for evolutionary dynamics and clonal analysis 16](#_Toc202780278)

[RNA isolation and RT-qPCR 16](#_Toc202780279)

[Generation of *trm* knockout in *A. baumannii* ATCC 17978 17](#_Toc202780280)

[REFERENCES FOR THIS DOCUMENT 19](#_Toc202780281)

1. **Supplementary Figures**

**Supplementary Figure S1.**

Morbidostat-based workflow of the experimental evolution of resistance to tigecycline (A,B) and colistin (C,D) in *A. baumannii* strains ATCC17978 (A,C) and BAA-747 (B,D).

**Figure S1A.** Experimental evolution of TGC-resistance in *A. baumannii* ATCC17978


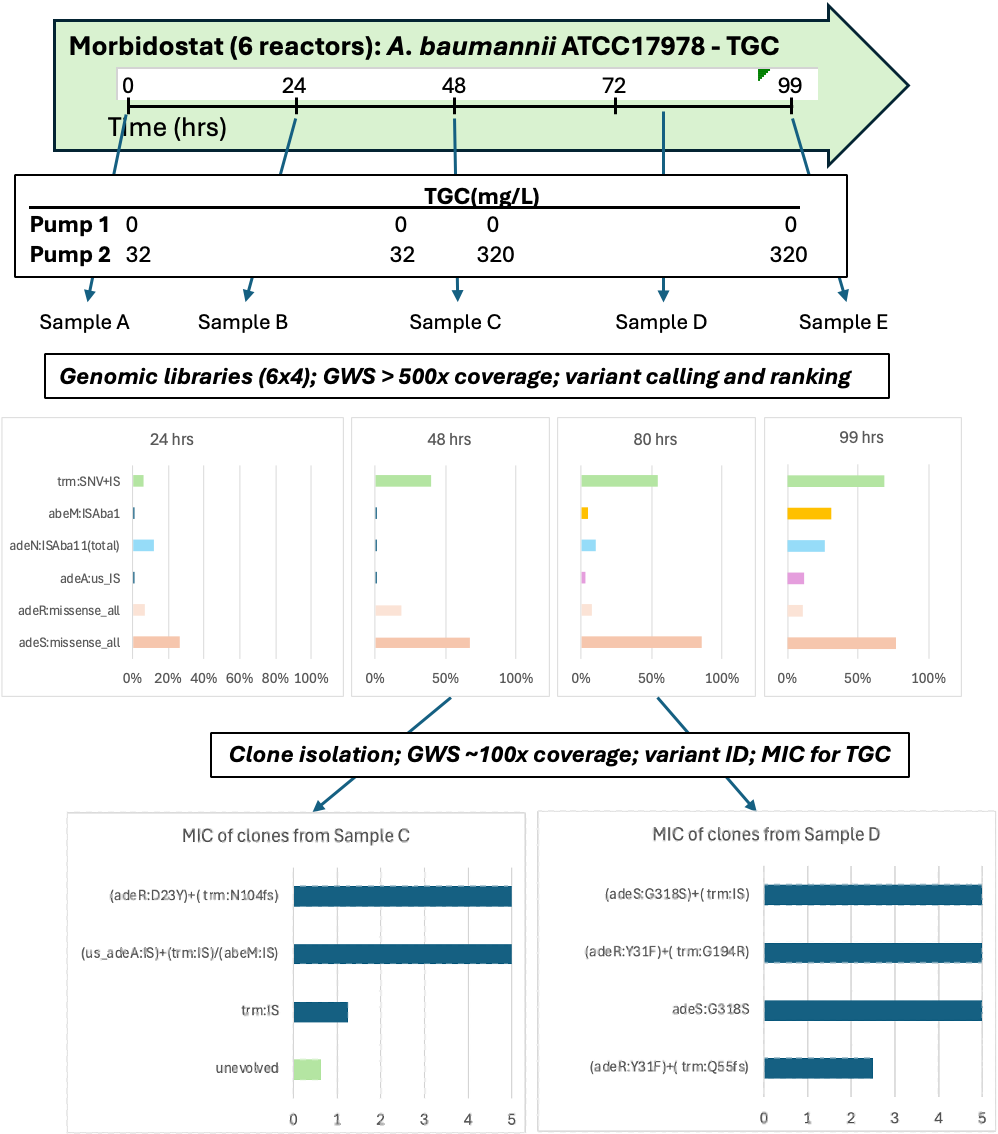
**Figure S1B.** Experimental evolution of TGC-resistance in *A. baumannii* BAA-747

**
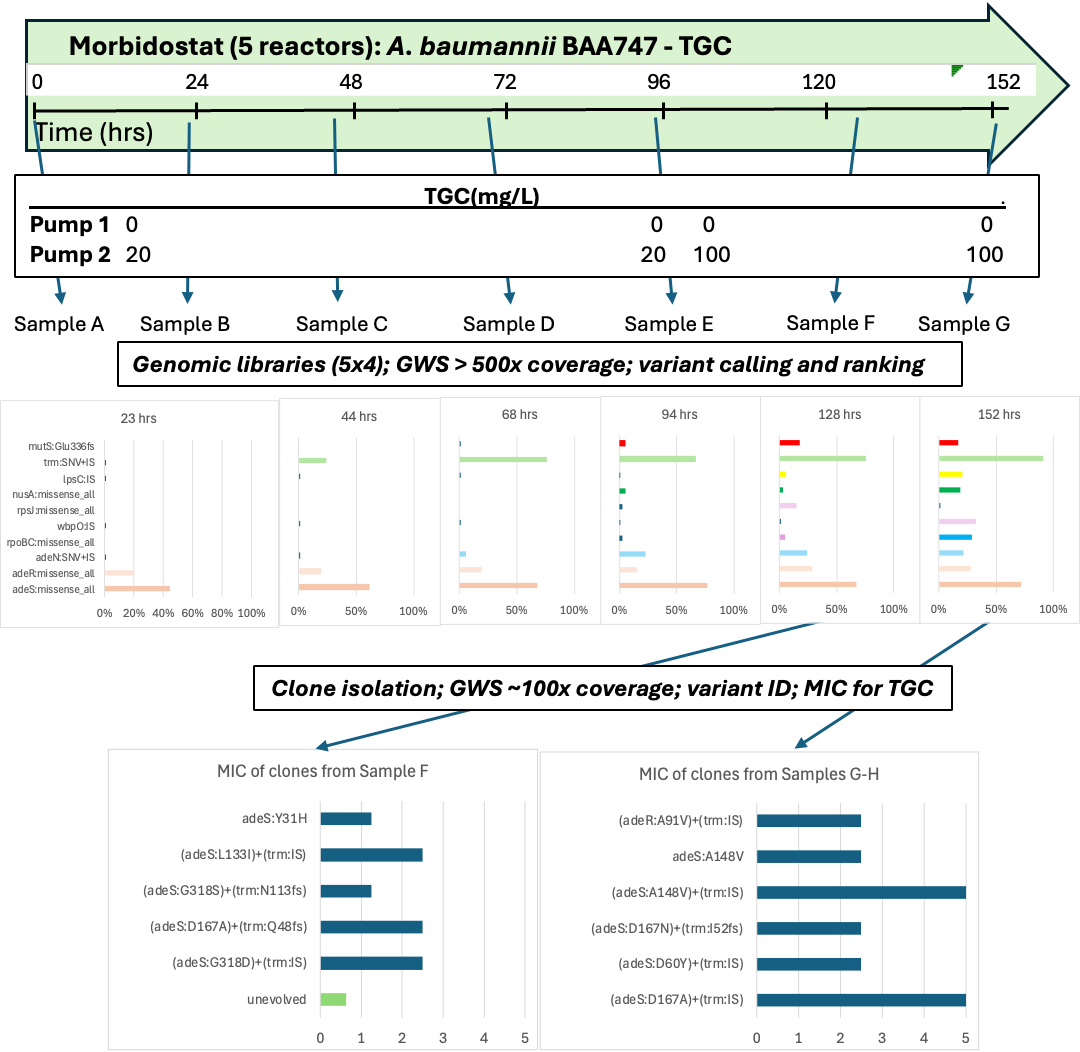
**

**Figure S1C.** Experimental evolution of COL-resistance in *A. baumannii* ATCC17978

**
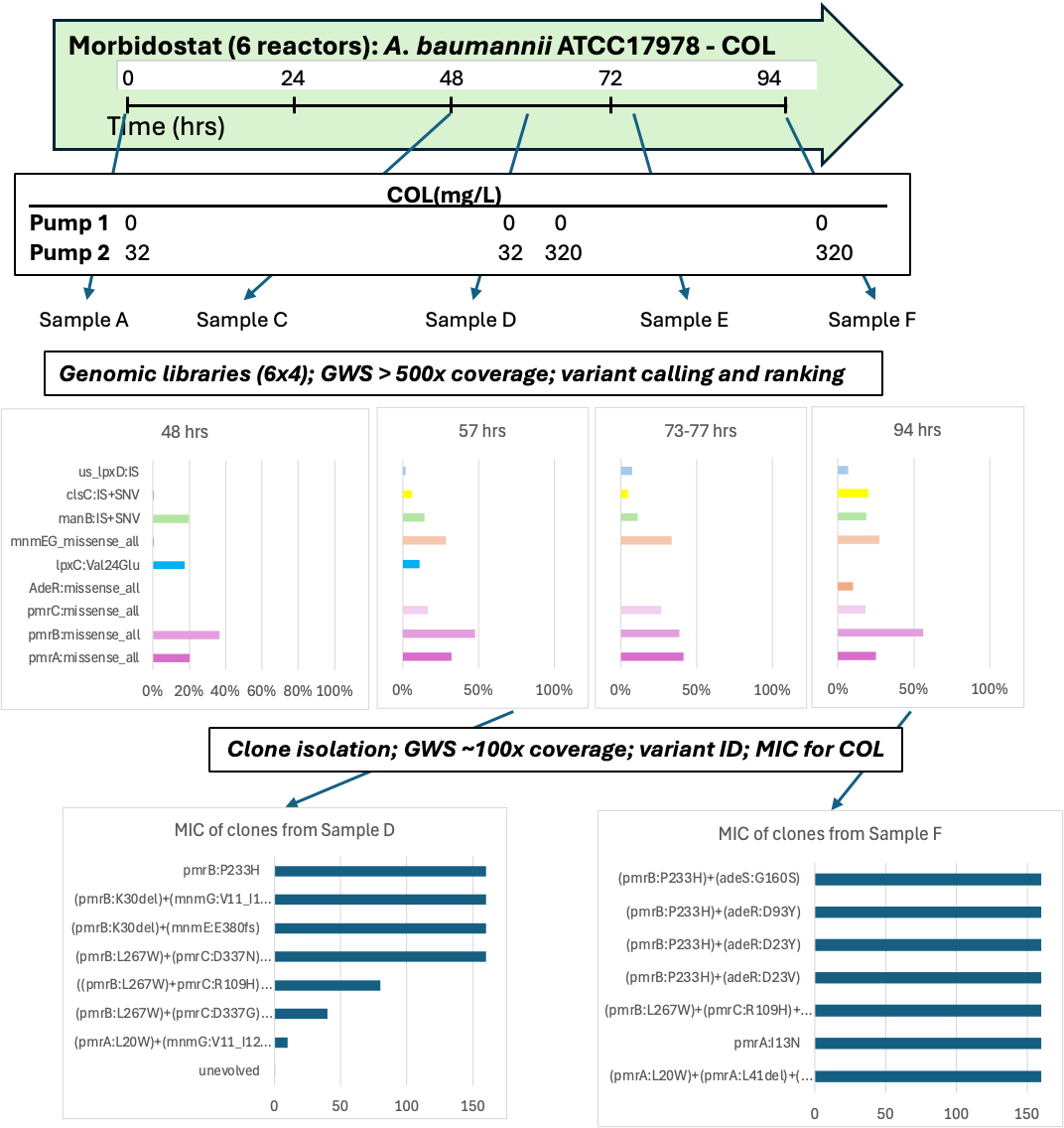
**

**Figure S1D.** Experimental evolution of COL-resistance in *A. baumannii* BAA-747

**
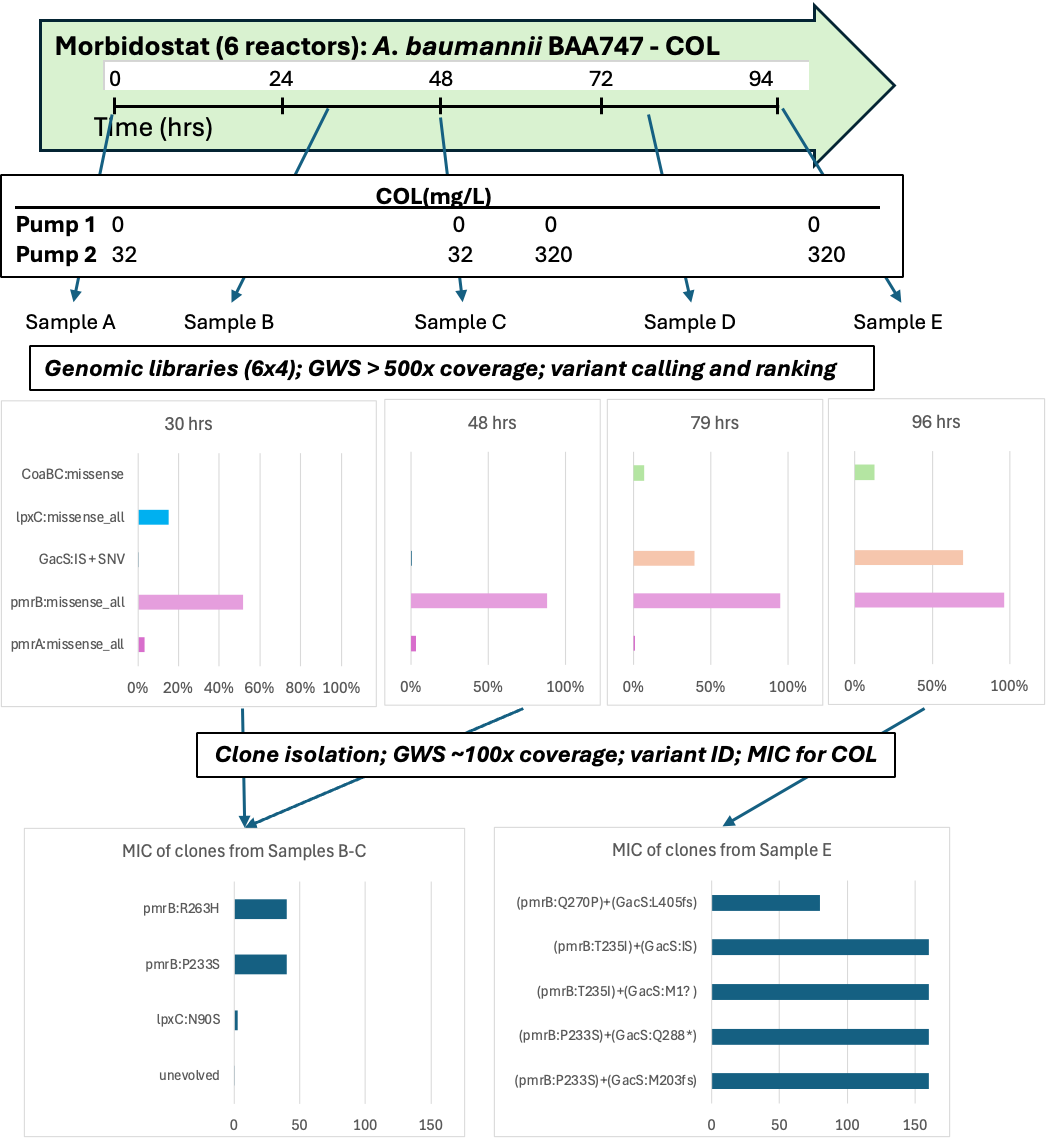
**

**Supplementary Figure S2.** Dynamics of major mutational variants in morbidostat-based evolution of resistance against TGC (A, B) and COL (C, D), in *A. baumannii*, ATCC 17978 (A, C) and BAA-747 (B, D).

**Figure S2A.** ***A. baumannii* ATCC17978/TGC:** Mutational dynamics in reactors R1–R6

**Figure S2B.** ***A. baumannii* BAA-747/TGC:** Mutational dynamics in reactors R1–R6

**Figure S2C.** ***A. baumannii* ATCC17978/COL:** Mutational dynamics in reactors R1–R6.

**Figure S2D.** ***A. baumannii* BAA-747/COL:** Mutational dynamics in reactors R1–R6

**Figure S2E.** ***A. baumannii* 17978 CST^R^ clones/TGC:** Mutational dynamics in reactors R1–R6 for A. baumannii ATCC 17978 clones which had previously been evolved in the presence of COL. R1& 2 inoculated with clone 2F2^COL^, R3& 4 inoculated with clone 2F1^COL^, R5& 6 inoculated with clone 2D3^COL^

Cumulative area plots are shown for potentially significant mutational variants (SNVs and IS insertions) observed in the evolving bacterial populations. Relative abundances of each selected variant (%) in each of the 6 reactors (R1-R6) are plotted vs evolution/sampling time (hrs). The shown subsets of variants (from the Supplementary Table S1 A,B,C,D) are limited to genes that fit to at least one of the two selection criteria: (i) feature more than one independent variant occurrence per gene (N_all_>1) with Amax≥10%; or (ii) feature a single variant occurrence per gene (N_all_=1) with Amax≥50%. The red line (at 100% corresponding to an average of 1 mutation per genome) helps to emphasize the emergence of double and triple mutants. Gene names and corresponding functions are explained below and in the Supplementary Table S1 providing complete tabular data.

**
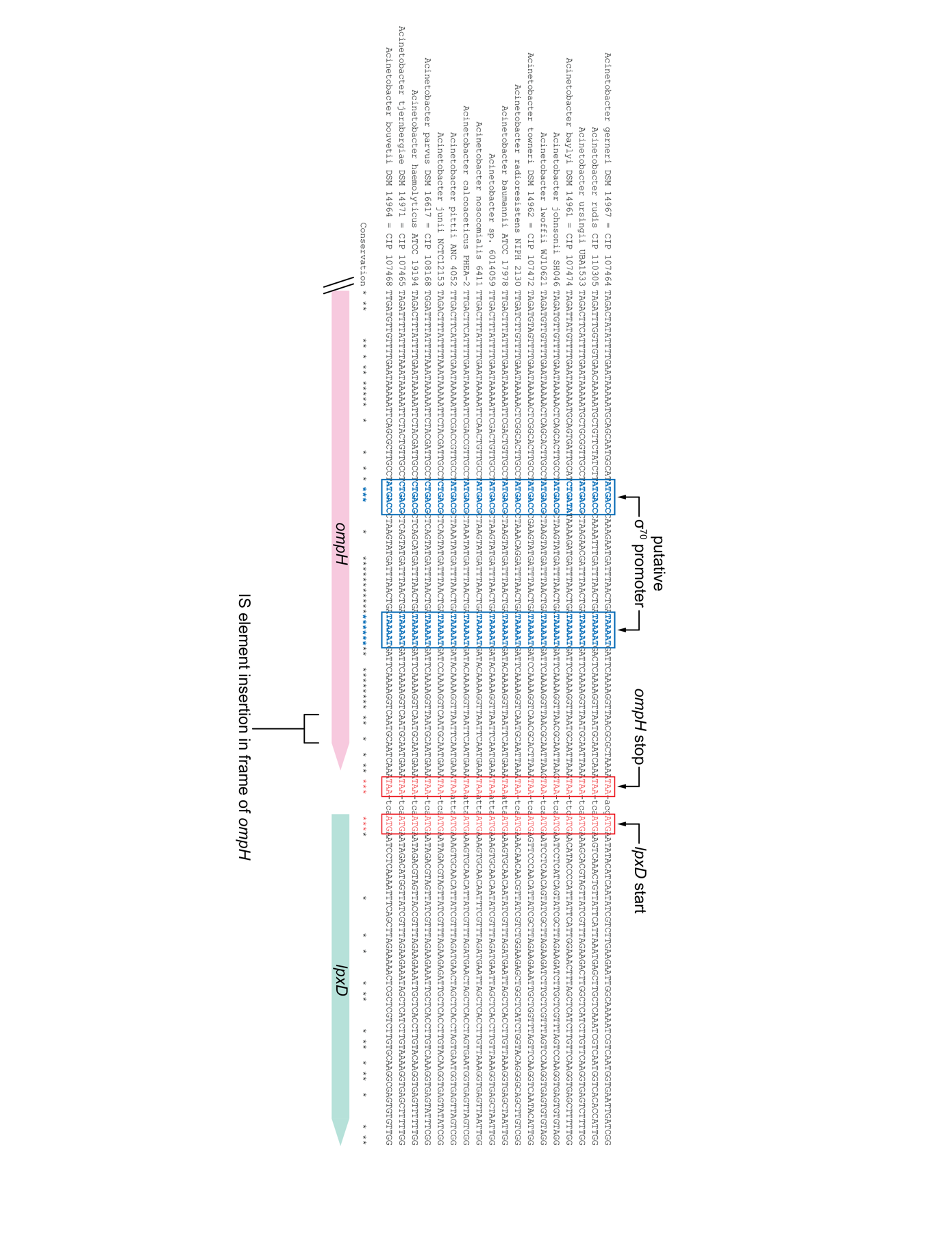
****Supplementary Figure S3**

**Impact of IS element insertions in *ompH* on *lpxD* transcription:** Multiple sequence alignment of the 3’ end of ompH and 5’ end of lpxD showing the consensus putative s70 promoter (blue box) and ompH stop and lpxD start codons. Cartoon schematics of the genes are shown below the nucleotide consensus track and the position of the IS element detected in a subset of clones from reactor 4 in ATCC 17978 evolved under COL pressure (4F1^COL^ and 4F3^COL^) is indicated.

**Supplementary Figure S4.**

Comparison of doubling times determined for drug-resistant clones isolated and characterized from the experimental evolution of A. baumannii strains ATCC17978 (A,B) and BAA-747 (C,D) for resistance against tigecycline (A,C) and colistin (B,D). Growth curves of selected clones determined (in duplicates) using microplate reader (OD_600_) over ~ 20 hrs in CA-MBH media (at 37^o^C, without drugs) were analyzed by the online tool “Dashing Growth Curves” ^1^, an interactive web application ( <http://dashing-growth-curves.ethz.ch/>) to extract maximal growth rates and doubling times.


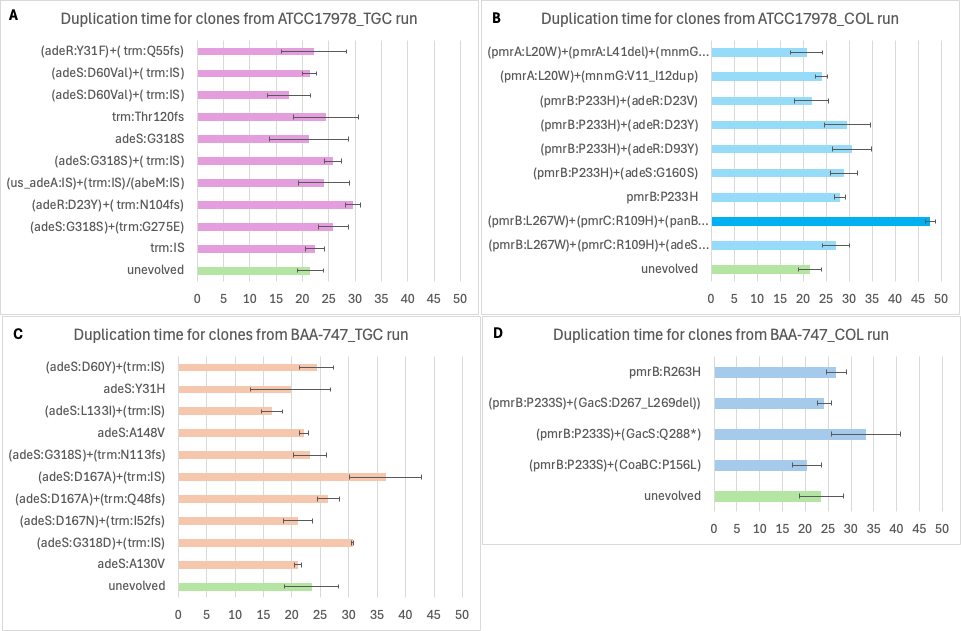


1. **Supplementary Methods**

# **Morbidostat setup and programming**

Our implementation of the morbidostat approach was described in our previous studies with GP-6, CIP and triclosan ^2,3^ ^4^. Briefly, we use a custom-engineered morbidostat device, which is a computer-controlled bundle of 6x(continuous culturing bioreactors) where the culture density (measured by OD600) is controlled by varying an antibiotic concertation in media. Automated regular dilutions (20% by volume, every 15 – 30 min) are performed using a controlled mixture of media from two feed bottles: (i) one, containing drug-free media and (ii) another one with drug-containing media. The composition of mixture is defined by the algorithm (fast growth – more drug/slow growth – less drug) enabling a gradually growing selective pressure driving the evolution of higher drug resistance. The detailed description of morbidostat implementation is provided on GitHub (<https://github.com/sleyn/morbidostat_construction>).

The main features of our morbidostat implementation include:

1. Six glass culture tubes containing 20 mL of media are used as bioreactors equipped with stirring bars for culture agitation and three needles for: (a) input of media and air, (b) waste removal and (c) sample collection.
2. Laser beam intensity is used to measure culture turbidity (optical density or OD at ~635 nm). The laser, photodiode and culture tube are installed in the 3D-printed rack.
3. A miniature air pump provides aeration and maintains air pressure for liquid displacement fitted with 0.22 µm air filters for sterility.
4. Drug-containing media and drug-free media are fed from two bottles connected via autoclavable silicon tubing to computer-controlled peristaltic pumps, each for one media type, followed by an array of 12 valves (2 valves per reactor, one for controlling flow of drug-free media and one – for drug-containing media).
5. The excess volume from the reactors (upon each dilution cycle) media is displaced to waste bottles by the constant air flow.
6. The temperature in morbidostat chamber is kept at 37^o^C with a heater controlled by thermal sensor.
7. All logic operations are controlled by an Arduino Mega 2560 Rev3 board. A user interface is implemented on PC using MegunoLink software (v.1.32.20005.0105; <https://www.megunolink.com/>).

The program logics and parameters of automated dilutions were as provided on GitHub (<https://github.com/sleyn/morbidostat_construction>)

# **Sequencing data analysis, variant calling and ranking**

The raw reads were processed as previously described ^3,4^. Briefly, upon adapter and quality trimming (BBDuk from BBTools suite v. 38.42, <https://sourceforge.net/projects/bbmap/>), the reads were aligned to reference genomes (BWA MEM v0.7.17 ^5^). LoFreq Viterbi module was used to refine alignment near indel regions ^6^. Base Quality Score Recalibration was made by Genome Analysis Toolkit (GATK) modules BaseRecalibrator and ApplyBQSR ^7^. Sites with high frequency variants were masked from BaseRecalibrator by calling variants in the alignment down-sampled to ~ 50x coverage with Picard tools v.2.2.1 (<https://broadinstitute.github.io/picard/>) based on the coverage estimates by mosdepth v. 0.2.6 ^8^. The VCF file for BaseRecalibrator “--known_sites” option was produced by GATK HaplotypeCaller. All other SAM and BAM files manipulations (sorting, indexing, merging and splitting) were performed with Samtools v1.9 ^5^. SNP and indels were identified using LoFreq v2.1.3 ^6^.

Insertion sequence (IS) elements rearrangements were identified by a developed iJump tool (<https://github.com/sleyn/ijump>). IS elements in the reference genomes were predicted using BLAST against ISFinder database ^9^. Predicted effects of mutations were assigned with SnpEff v4.3 ^10^. VCF files manipulations were performed with bcftools v1.3 ^11^. Copy number variation (CNV) were predicted by CNOGPro package v1.1 for R ^12^. Reference genomes were downloaded from PATRIC database ^13^. WGS data and assembly of all six unevolved clones (A1 – A6) of A. baumannii ATCC 17978 including variations compared to reference genomes were reported in the previous study ^3^. Nanopore reads were base-called using Guppy v.5.0.16 and aligned to reference with minimap2 v.2.17 ^14^. Large rearrangements were verified using Ribbon tool ^15^.

Ranking of the observed variants as significant was performed based on their statistics across all six reactors as described ^3,4^. Briefly, we initially prioritize variants reaching high frequency (or maximal abundance, in a population sample weighted on the total site abundance, ***A_max_***) in at least one sample across all 6 reactors. Then, for all genes implicated by at least one variant with ***A_max_*** ≥10% we consider all variants (non-preexisting, non-synonymous and with ***A_max_***≥2%). The second feature for further ranking of these initially prioritized genes, reflects the overall number of independently occurring variants per gene (***N_all_***). The latter combines a total number of distinct variants (***V***) with the number of reactors where each variant independently arises (***R***). Of those, the first parameter (***V***) is typically more significant, especially for disruptive mutations in coding regions (such as frameshifts, stop-gain and IS inserts). Therefore, we additionally prioritize the genes with high values of V>3, even with 2%≤***A_max_***<10% for all distinct variants, as long as their total abundance exceeds 10% in at least one sample. This approach typically implicates up to 8-10 genes with ***A_max_***≥50% and *N_all_*≥3 (up to 20 for disruptive mutations). In contrast to SNVs and even IS-inserts (where abundance determination is intrinsically less accurate), CNVs (large deletions and amplifications) cannot be accurately estimated in population WGS data. Besides, they cannot be unambiguously assigned to a specific gene, as they typically cover from 3 to more than 30 genes. Therefore, the prioritization (and interpretation) of the observed CNV events relies on the comparison with a list of genes implicated by other types of events. Identification of mutations in clones is straightforward. The observed variants typically match those from respective population data (although in rare cases additional mutations are observed), and their calculated abundance is usually >90%.

# **Population WGS data deconvolution for evolutionary dynamics and clonal analysis**

Optimization of clone selection aimed to maximally cover the diversity of individual variants was performed using Mixed Integer Programming to deconvolute population sequencing as described in^4^. The details of the algorithm and its implementation can be found at the GitHub page (<https://github.com/sleyn/subpop_decompose_mip>). The obtained subpopulation frequencies were used to choose the samples with the largest representation of the most prominent subpopulations that would allow us to maximize the odds and minimize the redundancy in their representation in clonal analysis.

# **RNA isolation and RT-qPCR**

RNA isolation from flash-frozen pellets was performed as described using a modified protocol of ^16^ with minor modifications. Briefly, cell pellets were resuspended in lysis buffer ( NaCl, 140 mM; EDTA, 14 mM; SDS, 6 % w/v) and mixed with Phenol_Chloroform: Iodoacetic acid (125: 24: 1, pH 4.5). Glass beads (acid washed, 500 uL) were added to each sample before being homogenized using a Bead Ruptor 12 (Omni Inc) for 2 minutes at 6 m/s 2 times. Subsequently, the samples were centrifuged (15 minutes, 16000 *g*, 4 °C) and the aqueous phase collected. Crude RNA was precipitated from this phase using sodium acetate (0.3 M final concentration) and isopropanol overnight at -20 °C. The precipitates from overnight were collected by centrifugation (30 minutes, 16000 *g*, 4 °C), washed 2 times in ice cold ethanol (70 %), dried in a laminar flow hood, and resuspended in nuclease free water. Coprecipitated DNA was removed using Baseline-ZERO^TM^ DNase (Lucigen) following the manufacturers protocol. Samples were further purified using the MEGAclear^TM^ Transcription Clean-Up Kit (Invitrogen). RNA concentration and quality was assessed via both nanodrop, using A260/280 and A260/230 ratios, and 1 % TAE agarose gel to monitor ribosomal RNA integrity. For samples which exhibited poor A260/A230 ratios, an additional round of ethanol precipitation was performed using the GlycoBlue^TM^ coprecipitant kit (Invitrogen), following the manufacturers instructions. Isolated RNA was frozen in liquid nitrogen and stored at -80 °C.

Complementary DNA was prepared from the isolated RNA using the High Capacity cDNA Reverse Transcription Kit (Thermo Fisher Scientific) following the manufacturers direction, with 200 ng of cDNA being prepared per clone per replicate. qPCR reactions were prepared using the PowerTrack^TM^ SYBR^TM^ Green Master Mix (Thermo Fisher Scientific) with 10 ng of prepared cDNA being used per gene for each sample and a final primer concentration of 400 nM. The reactions were performed on a Bio-Rad CFX384 Touch Real-Time PCR System with the following conditions: 95 °C for 10 minutes; 40 cycles of 95 °C for 15 s, 55 °C for 60 s. Threshold cycle (Ct) values were assigned for both the target gene, *mdtK*, and the reference gene, *gyrB* within CFX Manager^TM^ (Bio-Rad). Relative expression of *mdtK* and the associated standard deviations calculated for each clone using the 2^-ΔΔCT^ method ^17^. One-way ANOVA with Tukey’s HSD post-hoc testing was performed in R. The primers used are shown below.

Primers used

Target for the assessment of overexpression (designed in this study, efficiency ~100% as validated by serial dilution)

*adeA* FW primer: GTCCGCAAGTCGGAGGTATC

*adeA* RV primer: ATCGGCCTCAAAAGTCTCGG

*pmrC* FW primer: TCATTTGCAGTGGTCGGTGT

*pmrC* RV primer: CGCGCTGAATTTGATGAGCA

Housekeeping control for normalization^18^

*gyrB* FW primer: GATGATGCGCGTGAAGGTTT

*gyrB* RV primer: CATTGCTTGCTCTACCGCTG

# **Generation of *trm* knockout in *A. baumannii* ATCC 17978**

A knockout of the putative SAM-dependent methyltransferase, *trm*, was generated in the *A. baumannii* strain ATCC 17978 using a CRISPR-based gene editing approach as described by Wang *et al* ^19^.

Briefly, the spacer for targeting the PAM site within *trm* from *A. baumannii* was cloned into the guide RNA plasmid (pSGAb-Km) by Golden-Gate assembly, yielding the pSGAb-Km-trm plasmid.

The *trm* KO repair template was generated by performing overlap extension from two fragments belonging to the upstream and downstream regions of the gene, each ~ 1 kb in length. These fragments were generated by PCR using the Q5 High-Fidelity 2X Master Mix (NEB), with a 68 °C annealing temperature and 30 second extension time with genomic DNA from *A. baumannii* ATCC 17978 acting as the template. The overlap extension was alsoe performed using the same PCR mix, but with a 50 second elongation time. Successful fragment generation was validated prior to extraction from a 1% agarose TAE gel. This fragment was subsequently cloned into the pJET1.2 vector via blunt-end ligation using the CloneJET PCR Cloning Kit and following the manufacturers protocol. The pJET1.2-trmKO plasmid was subjected to site-directed mutagenesis to remove a 60 bp fragment remaining from the *trm* gene which was required to generate specific primers when amplifying the gene from the plasmid. The final repair template was amplified from this template by PCR and purified by gel extraction.

*A. baumannii* ATCC 17978 competent cells were prepared by washing overnight cultured with ice-cold water and ice-cold glycerol (10%). The CRISPR-Cas9 containing plasmid (240 ng) was transformed into *A. baumannii* by electroporation (2500 V, 25 µF, t_c_ 5 ms) and expression of the Cas9 was induced by addition of IPTG (1 M) to early log-phase culture the subsequent day after selection of positive transformants on apramycin (100 µg/mL) plates. Cas9 expressing *A. baumannii* were made electrocompetent as above and were subsequently transformed with pSGAb-Km-trm (195 ng) and the *trm* repair template (142 ng) by electroporation (2500 V, 25 µF, t_c_ 5 ms). Positive transformants were selected on apramycin (100 µg/mL) and kanamycin (50 µg/mL) plates and validated by PCR to check for *trm* deletion. Plasmids were cured by serial passaging in antibiotic free LB media at 37 °C overnight and streaking onto LB agar plates containing sucrose (5% w/v).

Primers used

*trm*_spacer FW primer TAGTAAACAGCGTTATGTACAGCA

*trm*_spacer RV primer AAACTGCTGTACATAACGCTGTTT

us_*trm* FW primer: GGTGGTGGATCCAGTCGCAGAGGTTGACTCCA

us_*trm* RV primer: TTCGATCGGCAACTGGTCTTCAAAGGAGAAAACCGACACTGA

ds_*trm* FW primer: AGTGTCGGTTTTCTCCTTTGAAGACCAGTTGCCGATCGAA

ds_*trm* RV primer: GGTGGTCTGCAGCTACTTTGATGGCGGCGTTG

*trm*_SDM FW primer: CATTTTTTAGGTATATCAGAAGGTGAGTGGATGTATAAAGTTATAGCG

*trm*_SDM RV primer: CGCTATAACTTTATACATCCACTCACCTTCTGATATACCTAAAAAATGGTGACTCATATA

*trm*_KO_check FW primer: GGTCGTAGGTTTCTAACACCG

*trm*_KO_check RV primer: CTGTAGCGATAGGCTGCTCTT

# **REFERENCES FOR THIS DOCUMENT**

1 Reiter, M. A. & Vorholt, J. A. Dashing Growth Curves: a web application for rapid and interactive analysis of microbial growth curves. *BMC Bioinformatics* **25**, 67, doi:10.1186/s12859-024-05692-y (2024).

2 Leyn, S. A. *et al.* Experimental evolution in morbidostat reveals converging genomic trajectories on the path to triclosan resistance. *Microb Genom* **7**, doi:10.1099/mgen.0.000553 (2021).

3 Zlamal, J. E. *et al.* Shared and Unique Evolutionary Trajectories to Ciprofloxacin Resistance in Gram-Negative Bacterial Pathogens. *mBio* **12**, e0098721, doi:10.1128/mBio.00987-21 (2021).

4 Leyn, S. A. *et al.* Two classes of DNA gyrase inhibitors elicit distinct evolutionary trajectories toward resistance in gram-negative pathogens. *npj Antimicrobials and Resistance* **2**, doi:10.1038/s44259-024-00021-y (2024).

5 Li, H. & Durbin, R. Fast and accurate short read alignment with Burrows-Wheeler transform. *Bioinformatics* **25**, 1754-1760, doi:10.1093/bioinformatics/btp324 (2009).

6 Wilm, A. *et al.* LoFreq: a sequence-quality aware, ultra-sensitive variant caller for uncovering cell-population heterogeneity from high-throughput sequencing datasets. *Nucleic Acids Res* **40**, 11189-11201, doi:10.1093/nar/gks918 (2012).

7 DePristo, M. A. *et al.* A framework for variation discovery and genotyping using next-generation DNA sequencing data. *Nat Genet* **43**, 491-498, doi:10.1038/ng.806 (2011).

8 Pedersen, B. S. & Quinlan, A. R. Mosdepth: quick coverage calculation for genomes and exomes. *Bioinformatics* **34**, 867-868, doi:10.1093/bioinformatics/btx699 (2018).

9 Siguier, P., Perochon, J., Lestrade, L., Mahillon, J. & Chandler, M. ISfinder: the reference centre for bacterial insertion sequences. *Nucleic Acids Res* **34**, D32-36, doi:10.1093/nar/gkj014 (2006).

10 Cingolani, P. *et al.* A program for annotating and predicting the effects of single nucleotide polymorphisms, SnpEff: SNPs in the genome of Drosophila melanogaster strain w1118; iso-2; iso-3. *Fly (Austin)* **6**, 80-92, doi:10.4161/fly.19695 (2012).

11 Li, H. A statistical framework for SNP calling, mutation discovery, association mapping and population genetical parameter estimation from sequencing data. *Bioinformatics* **27**, 2987-2993, doi:10.1093/bioinformatics/btr509 (2011).

12 Brynildsrud, O., Snipen, L. G. & Bohlin, J. CNOGpro: detection and quantification of CNVs in prokaryotic whole-genome sequencing data. *Bioinformatics* **31**, 1708-1715, doi:10.1093/bioinformatics/btv070 (2015).

13 Davis, J. J. *et al.* The PATRIC Bioinformatics Resource Center: expanding data and analysis capabilities. *Nucleic Acids Res* **48**, D606-D612, doi:10.1093/nar/gkz943 (2020).

14 Li, H. Minimap2: pairwise alignment for nucleotide sequences. *Bioinformatics* **34**, 3094-3100, doi:10.1093/bioinformatics/bty191 (2018).

15 Nattestad, M., Aboukhalil, R., Chin, C. S. & Schatz, M. C. Ribbon: intuitive visualization for complex genomic variation. *Bioinformatics* **37**, 413-415, doi:10.1093/bioinformatics/btaa680 (2021).

16 Rey, F. E. *et al.* Dissecting the in vivo metabolic potential of two human gut acetogens. *J Biol Chem* **285**, 22082-22090, doi:10.1074/jbc.M110.117713 (2010).

17 Schmittgen, T. D. & Livak, K. J. Analyzing real-time PCR data by the comparative C(T) method. *Nat Protoc* **3**, 1101-1108, doi:10.1038/nprot.2008.73 (2008).

18 Wong, M. H., Chan, B. K., Chan, E. W. & Chen, S. Over-Expression of ISAba1-Linked Intrinsic and Exogenously Acquired OXA Type Carbapenem-Hydrolyzing-Class D-ss-Lactamase-Encoding Genes Is Key Mechanism Underlying Carbapenem Resistance in Acinetobacter baumannii. *Front Microbiol* **10**, 2809, doi:10.3389/fmicb.2019.02809 (2019).

19 Wang, Y. *et al.* A highly efficient CRISPR-Cas9-based genome engineering platform in Acinetobacter baumannii to understand the H2O2-sensing mechanism of OxyR. *Cell Chemical Biology* **26**, 1732-1742. e1735 (2019).
